# Gα15 in early onset of pancreatic ductal adenocarcinoma

**DOI:** 10.1038/s41598-021-94150-3

**Published:** 2021-07-21

**Authors:** Giulio Innamorati, Thomas M. Wilkie, Giorgio Malpeli, Salvatore Paiella, Silvia Grasso, Borislav Rusev, Biagio Eugenio Leone, Maria Teresa Valenti, Luca dalle Carbonare, Samuele Cheri, Alice Giacomazzi, Marco Zanotto, Vanessa Guardini, Michela Deiana, Donato Zipeto, Michela Serena, Marco Parenti, Francesca Guzzi, Rita Teresa Lawlor, Giovanni Malerba, Antonio Mori, Giuseppe Malleo, Luca Giacomello, Roberto Salvia, Claudio Bassi

**Affiliations:** 1 Department of Surgical Sciences, Dentistry, Gynecology and Pediatrics, University of Verona, c/o GB Rossi General Hospital, P.le L.A. Scuro, 37134 Verona, Italy; 2 Pharmacology Department, UT Southwestern Medical Center, Dallas, TX USA; 3 ARC-Net Research Centre, University and Hospital Trust of Verona, Verona, Italy; 4 Department of Medicine and Surgery, University of Milano-Bicocca, Monza, Italy; 5 Department of Medicine, University of Verona, Verona, Italy; 6 Department of Neurosciences, Biomedicine and Movement Sciences, University of Verona, Verona, Italy

**Keywords:** Cancer, Cell biology, Molecular biology

## Abstract

The *GNA15* gene is ectopically expressed in human pancreatic ductal adenocarcinoma cancer cells. The encoded Gα15 protein can promiscuously redirect GPCR signaling toward pathways with oncogenic potential. We sought to describe the distribution of *GNA15* in adenocarcinoma from human pancreatic specimens and to analyze the mechanism driving abnormal expression and the consequences on signaling and clinical follow-up. We detected *GNA15* expression in pre-neoplastic pancreatic lesions and throughout progression. The analysis of biological data sets, primary and xenografted human tumor samples, and clinical follow-up shows that elevated expression is associated with poor prognosis for *GNA15*, but not any other *GNA* gene. Demethylation of the 5′ *GNA15* promoter region was associated with ectopic expression of Gα15 in pancreatic neoplastic cells, but not in adjacent dysplastic or non-transformed tissue. Down-modulation of Gα15 by shRNA or CRISPR/Cas9 affected oncogenic signaling, and reduced adenocarcimoma cell motility and invasiveness. We conclude that de novo expression of wild-type *GNA15* characterizes transformed pancreatic cells. The methylation pattern of *GNA15* changes in preneoplastic lesions coincident with the release a transcriptional blockade that allows ectopic expression to persist throughout PDAC progression. Elevated *GNA15* mRNA correlates with poor prognosis. In addition, ectopic Gα15 signaling provides an unprecedented mechanism in the early steps of pancreas carcinogenesis distinct from classical G protein oncogenic mutations described previously in *GNAS* and *GNAQ/GNA11*.

## Introduction

The incidence of pancreatic ductal adenocarcinoma (PDAC) is increasing as the disease remains a major challenge in oncology. Risk factors, such as chronic inflammation, stimulate oncogenic transformation over many years prior to presentation of symptoms. Deciphering the etiology of PDAC initiation and progression could identify early stage markers and treatment options crucial to extending survival.

Over 60 G protein coupled receptors (GPCRs) are expressed in PDAC and a third of these are orphan receptors without known ligands^1^. Their functions in PDAC are poorly characterized. GPCR signaling is transduced by heterotrimeric Gαβγ proteins. Four classes of Gα proteins, Gs, Gi, Gq, and G12, regulate distinct signaling pathways^2^. The large G proteins and small G proteins, such as Ras, share related structure and regulation of activity by a cycle of GTP binding and hydrolysis. Whole-exome and genome sequencing confirmed the importance of heterotrimeric G proteins in oncogenic signaling pathways^1^. The best-described molecular mechanism is a collection of amino acid substitutions in the GTP binding site that inhibits GTP hydrolysis analogous to activating mutations in KRAS. Driver mutations in *GNAS,* the gene encoding Gαs that activates adenylyl cyclase, are associated with cystic lesions termed intraductal papillary mucinous neoplasms (IPMNs), the second most frequent precursor of PDAC^3^ after pancreatic intraepithelial neoplasia (PanIN). The Gq class activates PLCβ and downstream calcium signaling. Activating mutations in the two ubiquitously expressed Gq class proteins, Gαq and Gα11, are the most common driver mutations in uveal melanoma^1^. Constitutive Gα activation hyperstimulates persistent downstream signaling, and this may explain why driver mutations in Gα family members are not tolerated in most cellular contexts but are advantageous in only a few well-defined neoplasia.

Gα15 is the most divergent Gα protein among human orthologs^2^. It promiscuously couples most GPCRs, thus redirecting signaling from other Gα pathways to activate calcium signaling^4,5^. Furthermore, once activated, Gα15 is poorly constrained by β-arrestin and RGS desensitization^6,7^. Possibly related to these signaling peculiarities, Gα15 expression is highly restricted, primarily to hematopoietic cells^8^. Gα15 may have evolved by providing a sensitive and potent signaling node in host cells dedicated to respond to a rapidly changing microenvironment.

In acute myeloid leukemia (AML), de Jonge et al*.* found that higher *GNA15* expression in the stem-cell enriched (CD34+) fraction was associated with poorer overall patient survival^9^. We found Gα15 was expressed in human PDAC biopsies, even after malignant cells were selectively expanded in nude mice^10^. In addition, Gα15 supported the growth of PDAC cell lines in suspension and nutrient deprivation^10^. Gα15 effectors include several therapeutic targets in clinical trials for PDAC, such as Ras, PI3K, PKCs^4^ and p53^11^. Ectopic Gα15 could hijack diverse GPCRs to activation of Ca^2+^ signaling and bypass β-arrestin dependent desensitization, obviating the need for classical activating mutations to sustain aberrant activity. Here we show that Gα15 de novo expression appears in the early phases of pancreas carcinogenesis, is highly specific of transformed cells, affects cellular signaling and migratory properties, and has prognostic implications for PDAC.

## Methods

### Ethics approval and consent to participate

The collection and use of human samples were performed in accordance with relevant guidelines and regulations and approved by the Ethical Committee of the University Hospital Trust of Verona (LCT001 and following amendments). Informed consent was obtained from all subjects.

### In situ* hybridization (ISH)*

RNAscope assay was performed on FFPE biopsies using RNAscope 2.5 HD Reagent Kit [RED 322,360, Advanced Cell Diagnostics (ACD), Hayward, CA] according to manufacturer instructions (https://acdbio.com). Briefly, 5 µm tissue sections were baked 1 h at 60 °C and deparaffinized with xylene and 100% ethanol. After 10 min incubation with H_2_O_2_ solution, target retrieval was performed using the Brown FS3000 food steamer for 30 min if tissue or 15 min if cells. Slices were dehydrated and incubated with protease plus at 40 °C for 15 min. The slides were then hybridized at 40 °C for 2 h with a probe for human Hs-GNA15 validated with cell lines previously characterized for *GNA15* expression by RT-PCR and western blot (Supp. Fig. S1)^10^ utilizing as a positive control probe Hs-UBC and as a negative control probe DapB. After hybridizations, slides were subjected to signal amplification using HD 2.5 detection kit, and hybridization signal was detected using a mixture of Fast-RED solutions A and B (1:60). Red dots reveal local amplification of the target mRNA. After counterstaining with Gill's hematoxylin, slides were dried in a 60 °C dry oven for 15 min and mounted with EcoMount. EM897L Biocare Medical, LLC). Sections were imaged and analyzed. Each sample was scored measuring the density of the dots in lesion area, values were confirmed comparing to a score (0 to 4) in blind by a pathologist.

### Gα15 downmodulation

Reagents for replication-deficient lentivirus were from Sigma-Aldrich. Lentiviral particles were produced by transient transfection of 3 µg of pLKO_IPTG1xLacO (SHGLY-NM) containing previously validated shRNA sequences^5^ and 3 µl of packaging mix (SHP001) into HEK293T cells in a 35 mm dish with Lipofectamine 2000 reagent (11,668,019, Life Technologies, Carlsbad, CA, USA), according to the manufacturer’s instructions. Viral supernatants were collected at 48 h, passed through a 0.2 μm filter and added to PT45 cells in the presence of 4 μg/mL hexadimethrine bromide (H9268) for 6 h followed by puromycin (P9620) selection 48 h post-infection.

### Gα15 knockout

CRISPR/Cas9-mediated GNA15 knockout was performed as previously described^7^. Briefly, Lipofectamine 2000 was used to transfect HEK293T cells, as above, with pSpCas9(BB)-2A-Puro(PX459)V2.0 (Addgene #62988) containing guides designed in CHOPCHOP and CRISPR design software and targeting GNA15 gene in the first (AGGATGAGAAGGCCGCCGCCCGG and CGGCCTTCTATCCTCCGTCAGG), and second (GTGCTCTTCCCGCTCTCGCCTGG) exons. Isolation of clonal cell lines was achieved with puromycin selection the third day after transfection followed by cloning into 96-well plates. DNA was isolated from single clones and 3 independent clones were selected for displaying premature stop codons in GNA15 sequence.

### Availability of data

1. GTEx Portal (https://gtexportal.org/home/gene/GNA15; January 2019), to study *GNA15* mRNA expression in normal human tissues.
2. Provisional dataset for Pancreatic Cancer (PAAD, https://portal.gdc.cancer.gov/projects/TCGA-PAAD) of The Cancer Genome Atlas (TCGA, https://www.cancer.gov/about-nci/organization/ccg/research/structural-genomics/tcga) queried through the cBioPortal^12^ (https://www.cbioportal.org/), to study *GNA15* mRNA expression in affected human tissues.
3. Broad Institute Cancer Cell Line Encyclopedia (CCLE^13^, https://portals.broadinstitute.org/ccle), to study *GNA15* mRNA expression, DNA methylation and their correlation in human cancer cell lines.
4. Data from three mRNA microarrays studies, to study *GNA15* gene expression in PDAC and healthy tissue^14–16^.
5. The Blueprint Epigenome Program (http://www.blueprint-epigenome.eu/), to study the correlation between *GNA15* mRNA expression levels and methylation at single base level in leukemic cells lineages. Gα protein mutation was analyzed in the GRCh38 Cosmic database v88^17^ on February 2019. Those who carried out the original analysis and collection of the data bear no responsibility for the further analysis or interpretation of it.
6. TCGA dataset LAML by MEXPRESS supported by the Common Fund of the Office of the Director of the National Institutes of Health, and by NCI, NHGRI, NHLBI, NIDA, NIMH.

### Statistical analysis

The statistical measurement values, when indicated, were presented as means ± SEM.

The statistical significance of differences in the measured mean frequencies between two experimental groups was calculated using the Student two-tailed t-test.

Experiments involving western blot and RT-PCR techniques were repeated n times as defined in the figure legends. Survival percentages were estimated using Kaplan–Meier methods, and survival curves were compared using the log-rank test.

Additional methods are described as supplementary information.

## Results

### *Gα15 *de novo expression distinguishes transformed pancreas from other cancers

**Figure 1:**
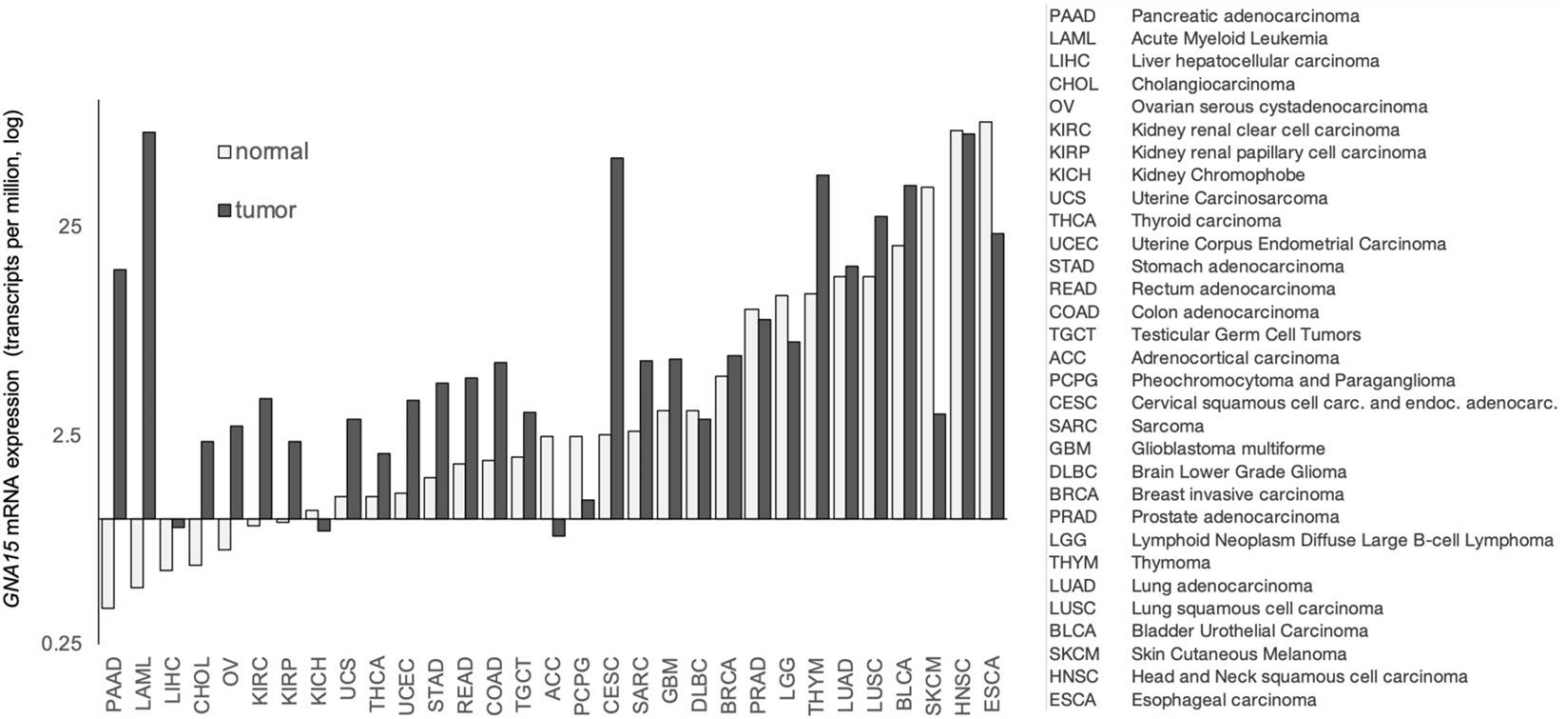
*GNA15* differential expression in normal tissue and cancer. Median gene expression across several tumor samples and paired normal tissues utilizing GEPIA2.

In mice^8^ and humans^4^, normal tissues predominately express *GNA15* in hematopoietic cells and skin progenitor cells. Systemic data extracted from the Genotype-Tissue Expression (GTEx) Project confirms these findings and includes two additional epithelia, esophagus mucosa and vagina (Supp. Fig. S2). By contrast, *GNA15* expression is lowest in pancreas and the entire gastrointestinal tract below the gastroesophageal junction. Characterization of *GNA15* expression in paired normal tissue and tumor samples revealed exocrine pancreas had among the highest differential induction of *GNA15* in cancer (Fig. 1).

Biomarker screens of paired samples of PDAC tumor versus adjacent healthy tissue shows *GNA15* is preferentially expressed in tumors of nearly all PDAC patients (Supp. Fig. S3)^14–16,18^.

**Figure 2:**
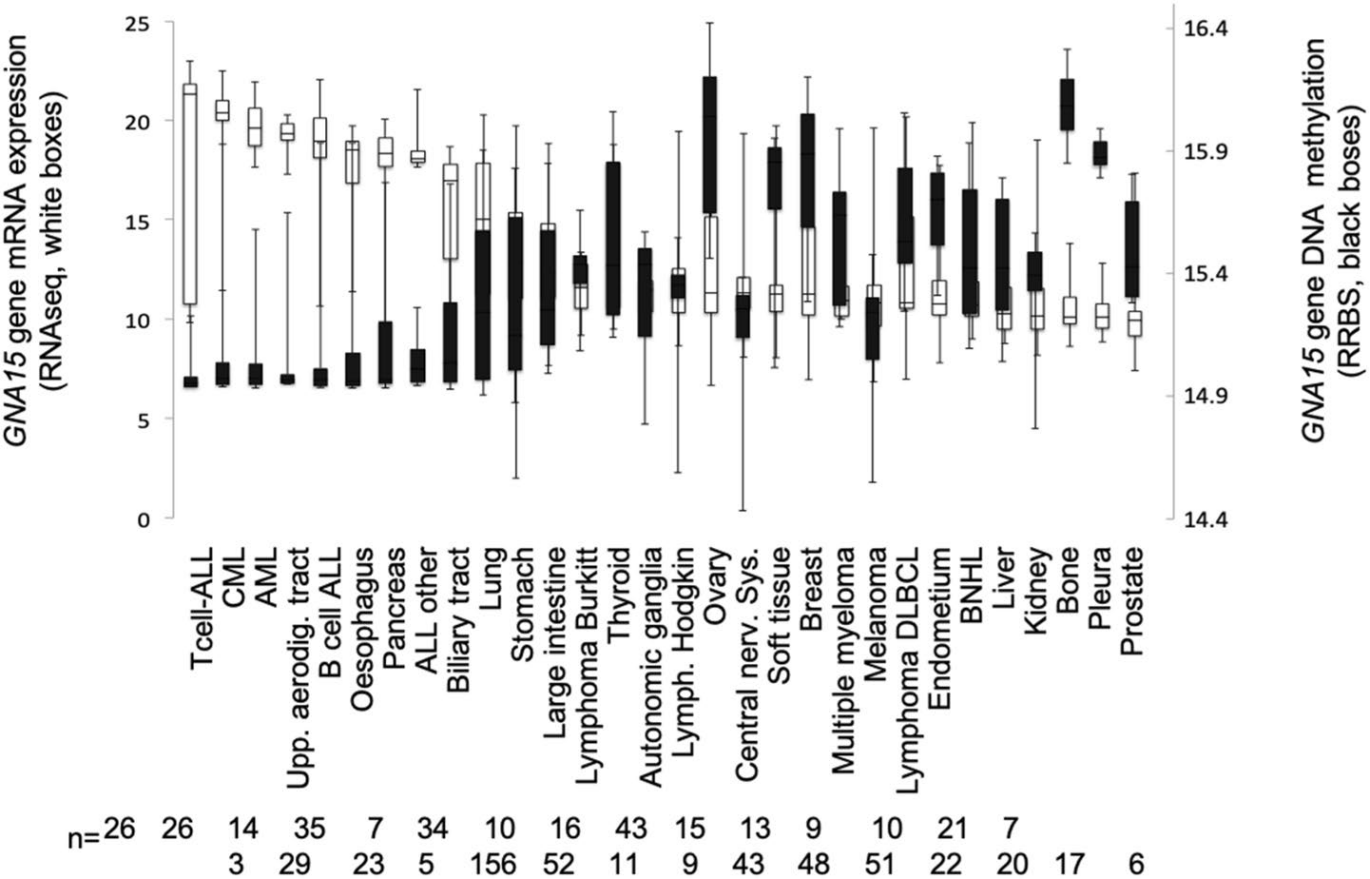
*GNA15* expression and methylation in cancer cell lines. *GNA15* mRNA expression (white bar showing RNAseq on the left axis) and DNA methylation (black bars, showing reduced representation bisulfite sequencing (RRBS) on the right axis) according to data from the Cancer Cell Encyclopedia. Among one thousand cancer cell lines, pancreas and biliary tract are the only normal tissues that do not express *GNA15* but the derived cancer cell lines had high *GNA15* expression levels, comparable to leukemia and upper aero-digestive tract. Box plots are shown as median and 25th and 75th percentiles. ALL acute lymphocytic leukemia, CML chronic myelocytic leukemia, AML acute myelocytic leukemia, DLBCL diffuse large B cell lymphoma, BNHL B cells non-Hodgkin lymphoma. The number below to the lineage name indicates how many cell lines are in the lineage.

Analysis of over a thousand cancer cell lines from the cancer cell encyclopedia (Fig. 2, open bars) corroborated our initial finding^10^. Pancreas, bile duct, and to a lesser extent, lung cancer cells are distinct among non-hematopoeitic lineages for ectopic *GNA15* expression at high levels similar to leukemia.

### GNA15 promoter demethylation correlates with Gα15 expression

**Figure 3:**
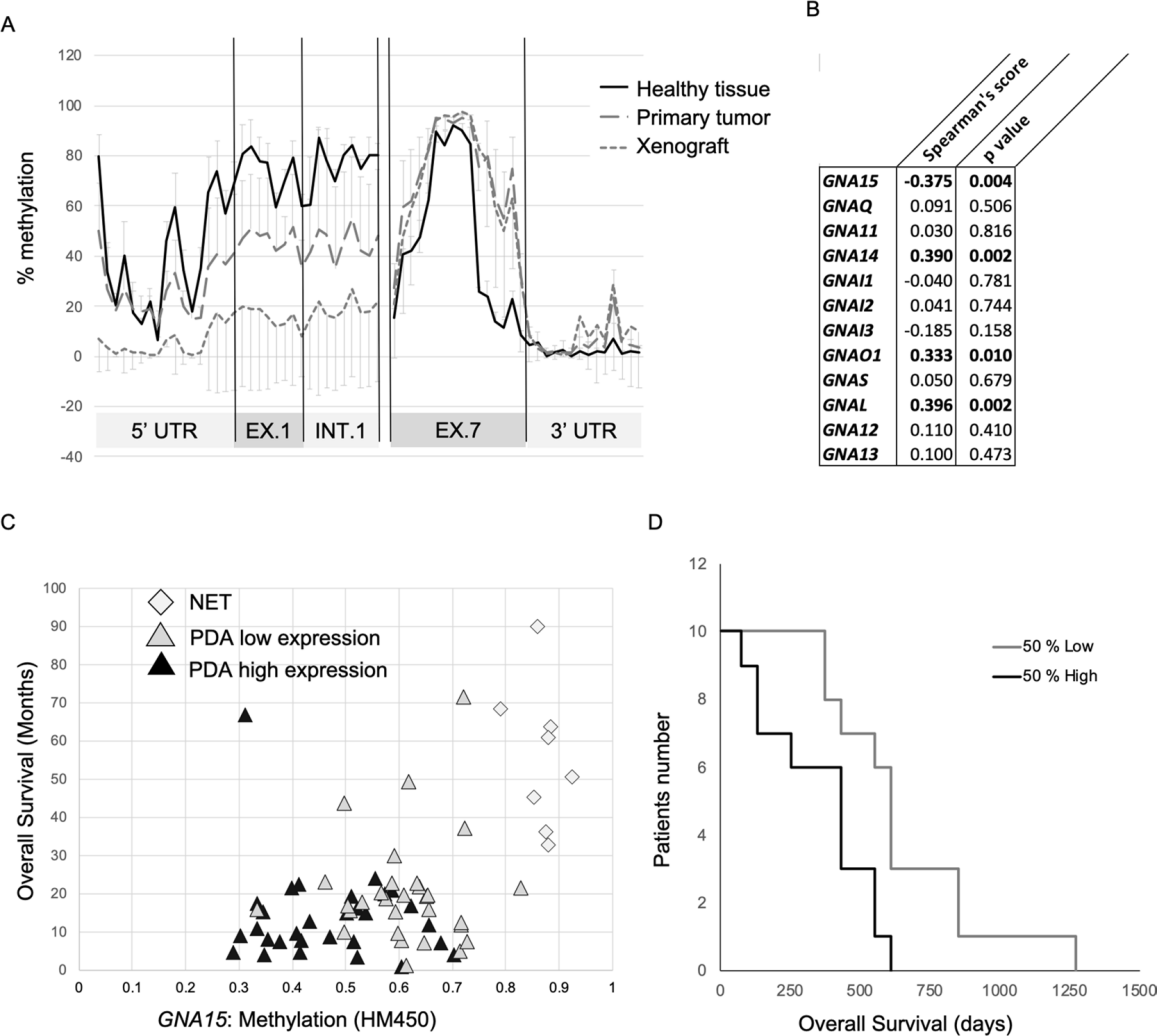
Demethylation, ectopic *GNA15* expression and survival. (**A**) *GNA15* gene methylation was mapped at single CpG in apparently normal tissue, and xenografted tumors, matched with primary tumors when available (gray bars represent standard deviation. n = 4 for normal tissues and primary tumors and n = 22 for xenografts). (**B**) Spearman’s correlation between Gα subunits expression levels and survival according to the TCGA-PAAD cohort (n = 59 samples). The isotypes associated to a significant p value (< 0.05) are in bold. No apparent correlation with tumor stages was found for any Gα gene. (**C**) The methylation level reported in the TCGA-PAAD court plotted against survival data distinguishing the NET and the PDAC patients further subdivided based on low (bottom 30%) vs high (top 30%) *GNA15* expression levels. (**D**) *GNA15* high expression is associated with poor survival. Kaplan survival curve in patients in the cytology group. All 20 patients were divided in two groups based on *GNA15* expression (log RNA Seq V2 RSEM, log-rank test; z = 2.67, p = 0.00754).

Methylation levels of representative CpGs throughout the *GNA15* gene correlated with its expression in hematopoietic cell types (Blueprint Epigenome Program, supp. Fig. S4A), acute myeloid leukemia (TCGA-LAML database, supp. Fig. S4B), PDAC derived cell lines (GTEx, Fig. 2) and TCGA-PAAD tumor samples (Supp. Fig. S4C). In particular, high *GNA15* mRNA expression corresponds to 7 CpGs hypomethylated near the promoter, and 4 CpGs hypermethylated at the 3′-end of *GNA15* (Supp. Fig. S4C with 9 of 11 CpGs with *p* < 0.001). In order to establish a direct correlation between differential *GNA15* methylation and transformation we sought to apply targeted bisulfite sequencing to genomic DNA derived from PDAC tumors, xenografts and adjacent apparently normal tissue. Mapping single CpGs confirmed the correlation between this methylation pattern and *GNA15* expression (Fig. 3A). Moreover, the same methylation pattern was observed in primary and xenograft tumors. A comparison to apparently normal tissue shows that methylation of exon 1 and intron 1 is reduced in primary tumors and xenograft samples. Demethylation of the promoter is more evident in the xenografted tumors than in the primary tumor. The effect could be explained by an increased proportion of cancer cells relatively to fibroblast in transplanted xenografts as a consequence of the loss of the large fibroblasts component when cancer cells are transplanted into the hosting tissue of the animal.

By contrast, exon 7 is highly methylated at its 5′-end in all samples (normal, primary tumor, and xenograft) while its 3′-end is equally hypermethylated in primary and xenograft tumors compared to normal pancreas.

### GNA15 ectopic expression and prognosis

Heterotrimeric G proteins are emerging key-players in pancreatic and other neoplasia^1,3^. Driver mutations are easily recognized in the α subunit of small and heterotrimeric G proteins because amino acid substitutions are strictly localized to residues that catalyze GTP hydrolysis (see “Introduction” section). However, a search of the cosmic database produced no evidence for driver mutations in *GNA15* or other Gq class members in PDAC (Supp. Table 1). We suggest that ectopic expression of wild-type Gα15 is sufficient to divert GPCR signaling towards pancreatic oncogenisis^4,10^ because wild-type Gα15 promiscuously and persistently^6^ couples most GPCRs and may activate Kras^19^, PKD1^10^ and other oncogenic drivers in PDAC progression.

The Cancer Genome Atlas program (TCGA) shows for pancreatic adenocarcinoma (PAAD) that among 16 Gα genes, only elevated expression of *GNA15* was associated with worse prognosis (Fig. 3B). By contrast, elevated expression of Gα subunit genes *GNAL, GNAO1,* and *GNA14* were correlated with increased survival after diagnosis. These associations are likely related to the abnormal *GNA15* expression in PDAC cancer cells, whereas *GNAL*, *GNAO1*, and *GNA14* are preferentially expressed in normal pancreas, which is progressively lost as transformed tissue prevails.

The PAAD dataset includes patients with PDAC and pancreatic neuroendocrine tumors (NETs^20^). NETs are produced in the pancreas by an oncogenic pathway distinct from PDAC. Transcriptome analysis clearly shows *GNA15* expression and methylation levels are reciprocally inverse in NET and PDAC (Supp. Fig. S5A). NETs do not appreciably express *GNA15* and have a more favorable prognosis. Plotting patient’s overall survival vs. *GNA15* methylation segregates NET from PDAC and, to a lesser extent, PDAC with low compared to high *GNA15* expression (Fig. 3C) yet retaining prognostic indications (Fig. 3D).

Elevated *GNA15* in PDAC was highly correlated with loss of *SMAD4* expression (Pearson: − 0.47, *p* = 9.2 e^−10^) and *S100A2* overexpression (Pearson: 0.46, *p* = 2.0 e^−9^). *SMAD4* and *S100A2* are considered the most promising prognostic indications based on single genetic markers^21^. Interestingly, *S100A2* and *GNA15* prognostic values are significant in a subset of PAAD patients initially diagnosed by cytology (e.g. peritoneal or pleural fluid, Supp. Fig. S6A–C) in samples from late stage disease that may be enriched in transformed cells. Altogether, these findings strongly suggest that distinctive epigenetic changes drive ectopic expression of Gα15 protein in PDAC and pancreas is the primary example within the entire organism where up-regulation of *GNA15* transcription marks oncogenic transformation. Therefore, it is crucial to determine the precise identity of neoplastic cells that ectopically express *GNA15* in PDAC.

### Gα15 ectopic expression in pancreas marks transformed cells

**Figure 4:**
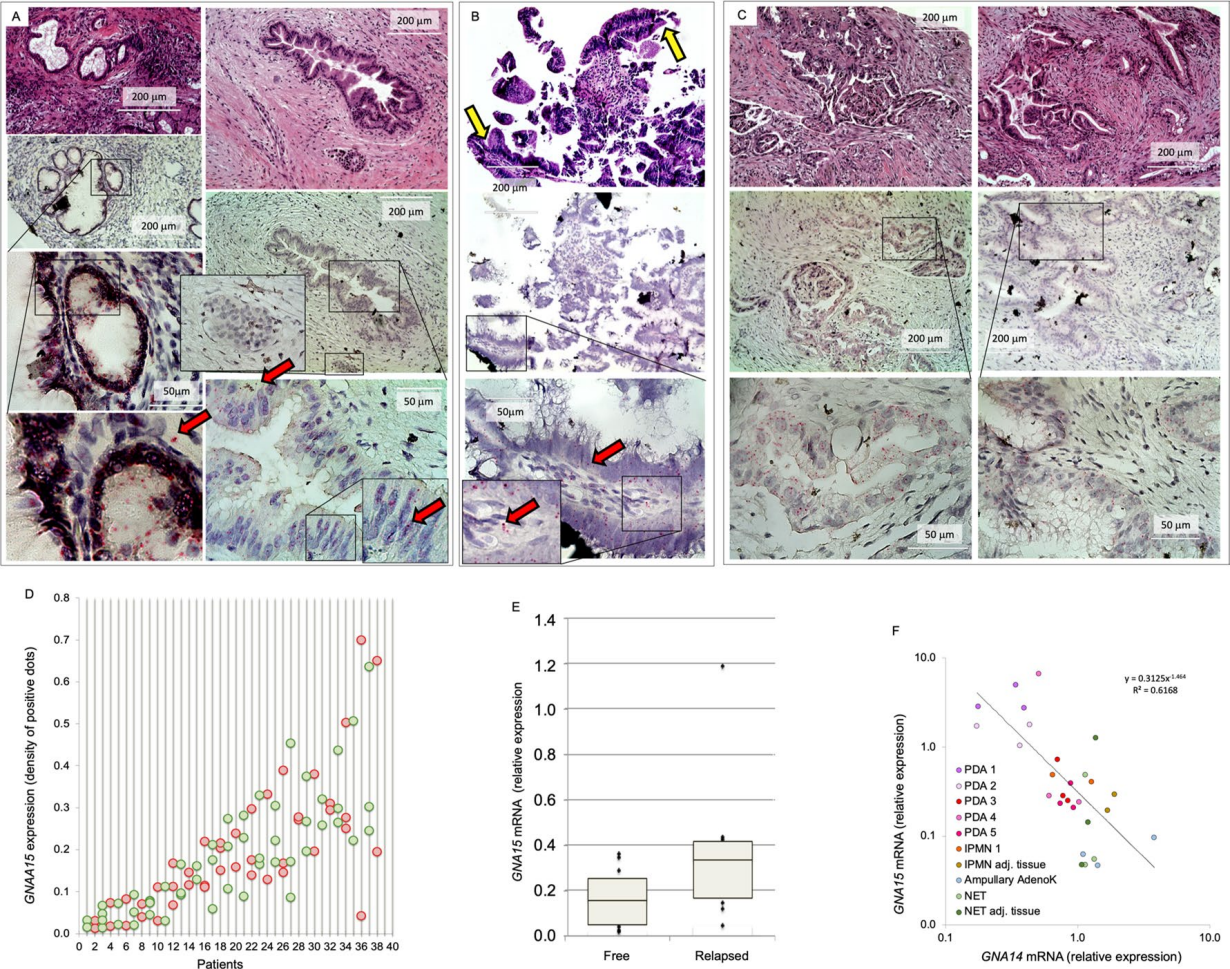
de novo *GNA15* gene expression in PDAC. (**A–C**) Human pancreas biopsies were analyzed by eosin hematoxylin (upper panels) or ISH (middle and lower panels) developed in Fast-RED (two examples are indicated by the red arrows) utilizing a *GNA15* specific probe in addition to positive and negative controls (Supp. Fig. S1). Preneoplastic and neoplastic lesions were evidently positive. A PanIN1 lesion is shown in panel A (left micrograph), and a PanIN2 (right micrograph). A superficial fragment of an IPMN is shown in panel B, the yellow arrows indicate papillary structures in the non invasive region. Panel C shows two examples of PDAC. (**D**) The expression level of *GNA15* was quantified by ISH. In the abscissa each number corresponds to a patient, and each circle represents the average value (dot density by ISH) of all lesions in a FFPE section, one circle per section, 2–4 sections per patient, 37 total patients. Red or green dots were used alternatively to differentiate each patient. (**E**) *GNA15* relative expression in the primary tumor was quantified by ISH and compared in patients that relapsed versus patients that remained disease free one year after pancreas resection. Box plots are shown as median and 25th and 75th percentiles: points are displayed as outliers if they are above or below the 1.5 times the interquartile range. A T-test was applied to compare the two groups, *p* < 0.05. (**F**) Tumor tissue fragments of resected pancreata were split in two parts. Half was analyzed by ISH to select for variable *GNA15* expression levels going from absent (apparently healthy tissue from NET patients) to high (PDAC with abundant positive lesions). The other half of the fragment was analyzed by TaqMan RT-PCR measuring *GNA15* and *GNA14* expression levels.

We analyzed *GNA15* mRNA expression by ISH among over 30 patients at the Verona Pancreas Center to determine in which cell types and stage of PDAC progression *GNA15* is ectopically expressed. Apparently healthy pancreatic tissue adjacent to transformed lesions was negative (Supp. Fig. S7A–C), consistent with prior studies^10^. Furthermore, *GNA15* was not induced in chronic pancreatitis nor in the fibrotic component or ductular structures in the desmoplastic reaction surrounding invasive lesions express *GNA15* (Supp. Fig. S8A). Infiltrating lymphocytes occasionally expressed *GNA15*, possibly depending on the activation state and the presence of plasma cells (Supp. Fig. S8B red dots identify *GNA15* mRNA ISH). Accordingly, the PAAD dataset shows no significant correlation between *GNA15* expression and lymphocyte infiltration stained by eosin hematoxylin or IHC (Supp. Fig. S4C). Neoplastic cells were consistently positive. Of note, PanIN (Fig. 4A) and IPMN (Fig. 4B) preneoplastic lesions expressed *GNA15* mRNA similar to virtually all lesions (Fig. 4C).

*GNA15* mRNA expression varied between patients but was relatively consistent within different specimens obtained from the same organ thus characterizing each patient (Fig. 4D). Frozen tissue samples adjacent to areas that produced FPPE sections with numerous neoplastic lesions and expressing high levels of *GNA15* mRNA were analyzed by western blot and compared to frozen samples adjacent to areas displaying apparently healthy tissue. A good correspondence was found between mRNA and protein expression (Supp. Fig. S9).

Follow-up data available for 20 PDAC patients (Supp. Table 2) show that one year after surgery half of the patients had relapsed. The average relative *GNA15* expression level was 0.37 in relapsed patients, compared to 0.16 of those that remained disease free (Fig. 4E), indicating that *GNA15* expression in transformed pancreas may offer indications on patient prognosis. Higher *GNA15* expression consistently predicted poorer survival (Supp. Fig. S10A), while no significant correlation was observed with Ca19.9 levels (Supp. Fig. S10B) or with other clinical parameters (Supp. Table 3), given the caveat of relatively few cases.

An inverse correlation in the PAAD dataset between the expression levels of *GNA15* and its closest homolog, *GNA14* (Spearman: − 0.26, *p* = 5.780e^−4^), was verified by RT-PCR on human frozen samples selected to include negative, intermediate and highly positive specimens on the basis of ISH analysis of adjacent fragments (Fig. 4F). Distinct samples from the same organ clustered together and thus distinguished each patient. This result suggests that the appearance of *GNA15,* but none of the other 15 *GNA* genes, could indicate progressive invasion into normal pancreas by PDAC (Supp. Fig. S10C).

We conclude that *GNA15* mRNA and protein expression in the pancreas is restricted to transformed cells, with minor contribution from infiltrating lymphocytes, occurs in the initial phases of PDAC progression (PanIN and IPMN), and may provide information about the development of the disease.

### Functional significance of Gα15 signaling

**Figure 5:**
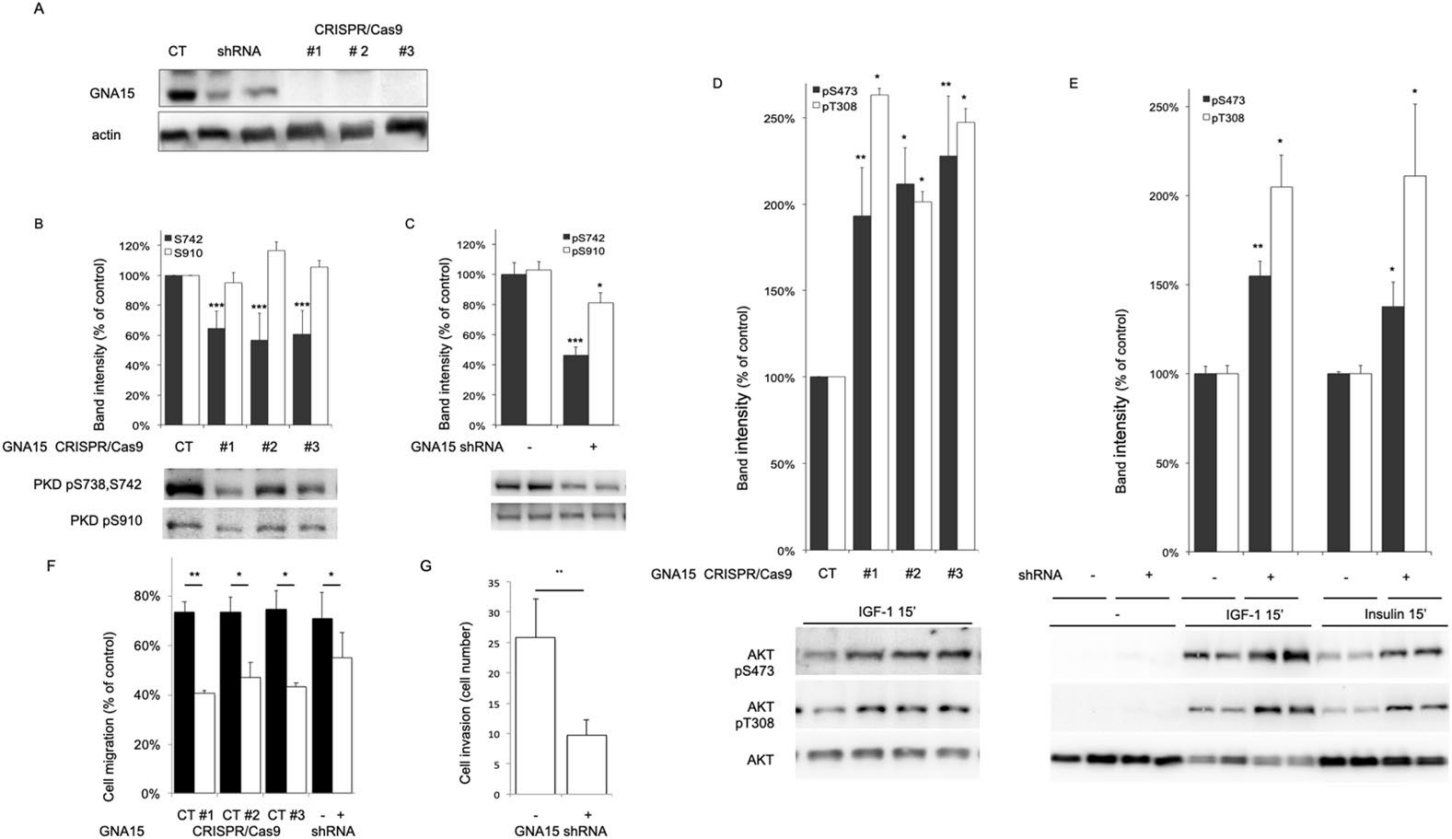
Functional consequences of Gα15 signaling. (**A**) In PT45 cells Gα15 was down-modulated by IPTG inducible shRNA expression or knocked out by CRISPR/Cas9 in all alleles of 3 independent clones, as confirmed by DNA sequencing. Protein down-modulation was confirmed by immunoblot as shown and quantified as 70% ± 9% of control (n = 4). (**B**) The impact of preventing Gα15 expression was evaluated by immunoblot analysis of phospho-amino acids diagnostic of the activation state of PKD1 (n = 10). (**C**) The impact of down-modulating Gα15 expression was evaluated as in B (n = 10). (**D**) The impact of preventing Gα15 expression in response to IGF was evaluated by immunoblot analysis of phospho-amino acids diagnostic of the activation state of AKT (n = 6). (**E**) The impact of down-modulating Gα15 expression in response to 100 ng/ml IGF and 100 nM insulin was evaluated as in D (n = 8). (**F**) PT45 cell motility assessed in scratch assays. Representative images are shown in Supp. Fig. S12. (**G**) PT45 cell invasion of collagen-coated membranes. Data are presented as mean ± SEM. Direct comparison between cells with *GNA15* downmodulated and controls was performed applying t-test and *, *p* < 0.05; **, *p* < 0.01; *** *P* < 0.0005. Extended blots are presented in Supp. Fig. S13.

In order to investigate the impact of Gα15 signaling, we further investigated PT45 cells^10,22^ as an experimental model system. Specific inhibition was achieved by two different approaches in PT45 PDAC cells. Complete knock out of *GNA15* gene was obtained by CRISPR/Cas9 editing. In parallel, validated shRNA sequences^10^ were engineered under the control of an IPTG inducible promoter and transduced in order to downmodulate *GNA15* expression in response to treatment (Fig. 5A).

We analyzed the activation state of selected effector kinases potentially downstream Gα15 signaling^4^ including protein kinase D1 (PKD1/PKCµ/PRKD1), a kinase involved in PDAC and downstream KRas-NF-κB signaling^23^. Upon *GNA15* depletion, the basal phosphorylation level of Ser738/Ser742 in the autoinhibitory domain of PKD1 was reduced. Yet, the phosphorylation of the regulatory Ser910 was not affected (Fig. 5B,C).

In intestinal epithelial cells, PKD1 mediates a negative feedback on the responsiveness of PI3K/AKT to extracellular stimuli^24^ and reprogramming towards stemness was observed in response to AKT downregulation^25^. Depletion of Gα15 expression up-regulated AKT response to Insulin-like growth factor (IGF-1). A similar increase of phosphorylation was observed for two different activation sites, Thr308 and Ser473, and by both approaches used to prevent Gα15 expression (Fig. 5D,E). The same effect on AKT after Gα15 depletion was obtained upon insulin stimulation (Fig. 5E). The activation state of ERK1/2, p38 and GSK remained substantially unaffected by Gα15 signaling under these conditions (Supp. Fig. S11). We conclude that Gα15 produces tonic activation of Ca^2+^ regulated PKD1 and affects AKT response to IGF receptor activation.

### The contribution of Gα15 signaling in cell motility

GPCRs ligands such as chemokines direct cell migration. We analyzed the impact of Gα15 signaling on the migratory properties of PT45 cells. Wound healing assays were sought to compare wild type vs. *GNA15* CRISPR/Cas9 knock out cells and untreated vs. shRNA down-modulated cells. Cell motility was reduced in both circumstances (Fig. 5F). The role of Gα15 in PT45 cell invasion was assayed using collagen-coated membranes (Fig. 5G). IPTG-induced shRNA expression knocked down *GNA15* expression and reduced cell invasion. These results support the possibility that Gα15 plays a role in the diffusion of transformed cells.

## Discussion

### De novo* ectopic GNA15 expression*

Somatic mutations are classically thought to drive cancer. Mutations that activate Gα subunits genes are oncogenic in a restricted set of neoplasias, such as Gαq/11-driven melanomas^1^ and Gαs-driven IPMNs in PDAC^3^. Unlike *GNAS,* no driver mutations are described for Gq-class genes in the pancreas (Supp. Table 1). Nevertheless, other mechanisms can activate oncogenic Gα signaling that more closely mimic signaling produced by physiological stimuli. For instance, down-regulation of the GPCR kinases (GRK) and regulators of G protein signaling (RGS) were recently associated with PDAC development^26,27^, implying that oncogenic GPCR signaling does not necessarily directly entail mutations.

We found ectopic expression of *GNA15* is typical of PDAC and occurs in transforming cells, rather than being contributed by stromal cells^10^; Fig. 4A–C, Supp. Fig S4,7,8). In prior biomarker screens of PDAC tumor tissue vs normal paired samples, *GNA15* was repeatedly identified among candidate genes (Supp. Figure 3) but was not further investigated, possibly because of relatively low expression restricted to a limited fraction of cancer cells within the tumor^14–16,18^. Among the four classes of heterotrimeric G protein alpha subunits^2^, Gα15 is unique in that it promiscuously redirects nearly all GPCRs to PLCβ activation and downstream calcium signaling^4^. In addition, Gα15 signaling is poorly controlled by β-arrestin and RGS dependent desensitization^6,7^. Ectopic expression of wild-type Gα15 in pancreas could subvert regulatory controls and could participate to influence the activity of downstream effectors, including PKC, PKD1, Src, PI3K, NF-kB, STAT and others that feed into Ras-MAPK activation and are relevant to PDAC^4,28^.

This intermediate step of ectopic Ga15 expression in PDAC initiation appears to be driven by altered DNA methylation of the *GNA15* gene. In transformed cells, hypomethylation shifts to the 5′ promoter region, apparently releasing a transcriptional blockade throughout PDAC progression.

Recently, the genomic region that includes *GNA15* was found to be differentially methylated by comparing EPCAM + / CD45- cells isolated from PDAC and normal pancreas^29^. In nearly pure PDAC and normal pancreatic cells, cytosine methylation in the 5′ side of the first intron occurred in the range of a beta value of 0.05–0.2 in PDAC and 0.6–0.8 in the normal pancreas. The two values are similar to those we found in xenografts and normal pancreas for the same DNA region. In addition, our data suggest that the beta value of 0.3–0.5 we observed in PDAC tissues, intermediate to those of xenografts and normal pancreas, is influenced by a variable cellularity of cancer cells. In the study by Espinet et al.^29^, an inverse correlation of approximately *r* = − 0.6 and a P value below 0.05 was also reported between the methylation of the first intron and the mRNA level, suggesting that the first intron includes regulatory sequences under epigenetic control.

The ectopic expression of Gα15 is unlikely a secondary effect of the poor vascularization that characterizes PDAC. In fact, areas affected by pancreatitis do not express *GNA15* despite the typical dysplastic reaction with massive deposition of stroma and the formation of small ducts^18^ (Supp. Fig. S7H,I). ISH of patient samples confirmed the data we obtained with xenografted mice showing that transformed cells express *GNA15* mRNA. In addition, tumor stroma was negative, with the exception of occasional infiltrated lymphocytes. Perhaps only activated lymphocytes express *GNA15*, as in most cases infiltrating lymphocytes are negative and there is no correlation reported in the TCGA-PAAD database.

In primary samples from human patients, *GNA15* mRNA expression varied from one patient to another, yet remained alike within multiple lesions collected from the same pancreas (Fig. 4E). *GNA15* expression invariably revealed the appearance of neoplastic cells. *GNA15* is first expressed in PanIN and IPMN, and at later stages of PDAC, elevated *GNA15* expression is correlated with poor survival. Altogether, our results suggest that ectopic expression of Gα15 may represent a marker of PDAC from early phases in tumor etiology. The cause-effect relationship remains to be understood.

Considering *GNA15* expression in PDAC is elevated above a negative background in the pancreas, the stringent correlation in expression linking *GNA15* with more established markers such as *SMAD4* and *S100A2* warrants additional research exploring clinical applications. *S100A2* was recognized as a prognostic marker in a large study showing the benefit of adjuvant therapy^30^. If Gα15 contributes to PDAC progression during asymptomatic phases, it is crucial to understand its role as transformed cells begin to engulf the pancreas and engraft outside the primary organ. Earlier diagnostic markers are desperately needed, as metastases render surgery palliative, rather than curative, even in the 20% of patients eligible for resection.

### The effects of ectopic GNA15 signaling

Gα15 ectopic expression in the pancreas could activate some of the most relevant drivers of PDAC such as oncogenic KRAS via the scaffold protein tetratricopeptide repeat 1 (TPR1^31^) and PKD1 via nPKC^32^. Gα15 induces PLCβ to cleave PIP_2_ producing the second messengers DAG and IP_3_ that synergistically activate protein kinase C (PKC)^4^. nPKCs phosphorylate Ser738/Ser742 of the PKD1 activation domain. Reduced phosphorylation at these positions upon *GNA15* silencing confirmed the impact of Gα15 on PDAC cells consistent with reduced PLCβ signaling and PKC activity (Fig. 5B,C).

PKD1 is expressed by pancreas cells and its promoter is a target of the KRAS-NF-κB pathway^23^. PKD1 protein is activated by KRAS and growth factors during the initiation of PDAC. Animal models showed PKD1 controls KRAS-dependent progression from metaplasia to preneoplastic lesions^32^ although we did not find a significant impact on the basal level of MAPK activation. PKD1 reduced activation was matched by the sensitization of AKT in response to IGF1 and insulin (Fig. 5D,E), as previously described in intestinal non-transformed IEC18 cells as a consequence p85α phosphorylation^24^. AKT signaling is widely considered carcinogenic and pro-tumorigenic, but it cannot be assumed to apply to all three isoforms and to all cancer cell types. For instance, in breast cancer, AKT 1 and AKT3 were reported to suppress motility^33^, invasion^34^ and metastasis^35^. Our study was limited to analyzing acute AKT activation in response to IGF since the basal activation state remained below the sensitivity threshold for both phosphorylation sites analyzed. Therefore, no direct inference can be made about AKT activity level and motility. The correlation between elevated expression of Gα15, early relapse, and poor survival, may result from supporting progenitor stages previously characterized by reduced AKT activity^36^. Our findings are consistent with data showing that silencing IGF/Insulin receptors sustained pluripotency of pancreatic progenitors^37^ and that silencing AKT, especially isoforms 1 and 3, evoked reprogramming of human pancreatic cancer cells towards stem cell-like properties^25^. Under the hypoxic conditions of the tumor niche, IGF stimulation could damage PDAC cells^38^. The upregulation of IGF stimulation could as well represent a compensatory effect. Similarly, the ablation of oncogenic drivers, such as KRAS^G12D^ or cMyc, produced compensatory autocrine upregulation of IGFR-AKT pathway that was interpreted as crucial for resistance of dormant cancer cells and recurrence^39^. Specifically designed studies are certainly required to verify if Gα15 may contribute to the dynamic modulation of AKT signaling to support slow cycling and invasive progenitor cells from the early phases of PDAC progression. Further studies are necessary to assess the contributions of insulin and IGF-1 under experimental settings that more closely reproduce the actual microenvironment present in patients with pancreatic cancer. Our findings indicate multiple variables must be considered, such as the specific AKT isoforms, their subcellular compartmentalization, tyrosin kinase receptor activation and the metabolic state of the cell.

## Conclusion

In conclusion, the reallocation of methylated cysteines in the *GNA15* gene is associated with the presence of Gα15 in PDAC, starting with precursor lesions and continuing through all stages of PDAC. *GNA15* expression is detectable substantially above a negative background, concomitant with the expression of prognostic markers of aggressive progression and poorer survival. Loss-of-function studies in PDAC cell lines showed Gα15 sustains oncogenic signaling and cell motility. Our data support a rationale for further investigating the potential of *GNA15* as an early marker of PDAC transformation and prognosis.

## Supplementary Information

Supplementary Information.

## Abbreviations

GPCR: G protein coupled receptor
IGF-1: Insulin-like growth factor
IPMN: Intraductal papillary mucinous neoplasm
PanIN: Pancreatic intraepithelial neoplasia
PDAC: Pancreatic ductal adenocarcinoma
PKC: Protein kinase C
PKD1: Protein kinase D

## References

[1] O'Hayre M, Vazquez-Prado J, Kufareva I, Stawiski EW, Handel TM, Seshagiri S (2013). The emerging mutational landscape of G proteins and G-protein-coupled receptors in cancer. Nat. Rev..

[2] Wilkie TM, Gilbert DJ, Olsen AS, Chen XN, Amatruda TT, Korenberg JR (1992). Evolution of the mammalian G protein alpha subunit multigene family. Nat. Genet..

[3] Innamorati G, Wilkie TM, Kantheti HS, Valenti MT, Dalle Carbonare L, Giacomello L (2018). The curious case of Galphas gain-of-function in neoplasia. BMC Cancer.

[4] Giannone F, Malpeli G, Lisi V, Grasso S, Shukla P, Ramarli D (2010). The puzzling uniqueness of the heterotrimeric G15 protein and its potential beyond hematopoiesis. J. Mol. Endocrinol..

[5] Wu D, LaRosa GJ, Simon MI (1993). G protein-coupled signal transduction pathways for interleukin-8 1. Science.

[6] Innamorati G, Giannone F, Guzzi F, Rovati GE, Accomazzo MR, Chini B (2009). Heterotrimeric G proteins demonstrate differential sensitivity to beta-arrestin dependent desensitization. Cell Signal.

[7] Masuho I, Balaji S, Muntean BS, Skamangas NK, Chavali S, Tesmer JJG (2020). A global map of G protein signaling regulation by RGS proteins. Cell.

[8] Wilkie TM, Scherle PA, Strathmann MP, Slepak VZ, Simon MI (1991). Characterization of G-protein alpha subunits in the Gq class: Expression in murine tissues and in stromal and hematopoietic cell lines. Proc. Natl. Acad. Sci. USA.

[9] de Jonge HJ, Woolthuis CM, Vos AZ, Mulder A, van den Berg E, Kluin PM (2011). Gene expression profiling in the leukemic stem cell-enriched CD34+ fraction identifies target genes that predict prognosis in normal karyotype AML. Leukemia.

[10] Giovinazzo F, Malpeli G, Zanini S, Parenti M, Piemonti L, Colombatti M (2013). Ectopic expression of the heterotrimeric G15 protein in pancreatic carcinoma and its potential in cancer signal transduction. Cell. Signal..

[11] Kim DM, Choi SH, Yeom YI, Min SH, Kim IC (2016). Genome-scale functional analysis of the human genes modulating p53 activity by regulating MDM2 expression in a p53-independent manner. Biochem. Biophys. Res. Commun..

[12] Gao J, Aksoy BA, Dogrusoz U, Dresdner G, Gross B, Sumer SO (2013). Integrative analysis of complex cancer genomics and clinical profiles using the cBioPortal. Sci. Signal.

[13] Safikhani Z, Smirnov P, Thu KL, Silvester J, El-Hachem N, Quevedo R (2017). Gene isoforms as expression-based biomarkers predictive of drug response in vitro. Nat. Commun..

[14] Badea L, Herlea V, Dima SO, Dumitrascu T, Popescu I (2008). Combined gene expression analysis of whole-tissue and microdissected pancreatic ductal adenocarcinoma identifies genes specifically overexpressed in tumor epithelia. Hepatogastroenterology.

[15] Segara D, Biankin AV, Kench JG, Langusch CC, Dawson AC, Skalicky DA (2005). Expression of HOXB2, a retinoic acid signaling target in pancreatic cancer and pancreatic intraepithelial neoplasia. Clin. Cancer Res. Off. J. Am. Assoc. Cancer Res..

[16] Pei H, Li L, Fridley BL, Jenkins GD, Kalari KR, Lingle W (2009). FKBP51 affects cancer cell response to chemotherapy by negatively regulating Akt. Cancer Cell.

[17] Sondka Z, Bamford S, Cole CG, Ward SA, Dunham I, Forbes SA (2018). The COSMIC cancer gene census: Describing genetic dysfunction across all human cancers. Nat. Rev..

[18] Iacobuzio-Donahue CA, Maitra A, Shen-Ong GL, van Heek T, Ashfaq R, Meyer R (2002). Discovery of novel tumor markers of pancreatic cancer using global gene expression technology. Am. J. Pathol..

[19] Liu AM, Lo R, Guo EX, Ho MK, Ye RD, Wong YH (2011). Galpha16 interacts with tetratricopeptide repeat 1 (TPR1) through its beta3 region to activate Ras independently of phospholipase Cbeta signaling. BMC Struct. Biol..

[20] Peran I, Madhavan S, Byers SW, McCoy MD (2018). Curation of the pancreatic ductal adenocarcinoma subset of the cancer genome atlas is essential for accurate conclusions about survival-related molecular mechanisms. Clin. Cancer Res..

[21] Collisson EA, Bailey P, Chang DK, Biankin AV (2019). Molecular subtypes of pancreatic cancer. Nat. Rev. Gastroenterol. Hepatol..

[22] Kalthoff H, Roeder C, Humburg I, Thiele HG, Greten H, Schmiegel W (1991). Modulation of platelet-derived growth factor A- and B-chain/c-sis mRNA by tumor necrosis factor and other agents in adenocarcinoma cells. Oncogene.

[23] Doppler H, Panayiotou R, Reid EM, Maimo W, Bastea L, Storz P (2016). The PRKD1 promoter is a target of the KRas-NF-kappaB pathway in pancreatic cancer. Sci. Rep..

[24] Ni Y, Sinnett-Smith J, Young SH, Rozengurt E (2013). PKD1 mediates negative feedback of PI3K/Akt activation in response to G protein-coupled receptors. PLoS ONE.

[25] Arasanz H, Hernandez C, Bocanegra A, Chocarro L, Zuazo M, Gato M (2020). Profound reprogramming towards stemness in pancreatic cancer cells as adaptation to AKT inhibition. Cancers.

[26] Liu WJ, Zhou L, Liang ZY, Zhou WX, You L, Zhang TP (2018). High expression of GRK3 is associated with favorable prognosis in pancreatic ductal adenocarcinoma. Pathol. Res. Pract..

[27] Ocal O, Pashkov V, Kollipara RK, Zolghadri Y, Cruz VH, Hale MA (2015). A rapid in vivo screen for pancreatic ductal adenocarcinoma therapeutics. Dis. Model. Mech..

[28] Huang YS, Hu CH, Tseng WY, Cheng CH, Stacey M (2017). Activation of adhesion GPCR EMR2/ADGRE2 induces macrophage differentiation and inflammatory responses via Galpha16/Akt/MAPK/NF-kappaB signaling pathways. Front. Immunol..

[29] Espinet E, Gu Z, Imbusch CD, Giese NA, Buscher M, Safavi M (2021). Aggressive PDACs show hypomethylation of repetitive elements and the execution of an intrinsic IFN program linked to a ductal cell of origin. Cancer Discov..

[30] Bachet JB, Marechal R, Demetter P, Bonnetain F, Cros J, Svrcek M (2013). S100A2 is a predictive biomarker of adjuvant therapy benefit in pancreatic adenocarcinoma. Eur. J. Cancer.

[31] Liu AM, Lo RK, Lee MM, Wang Y, Yeung WW, Ho MK (2010). Galpha16 activates Ras by forming a complex with tetratricopeptide repeat 1 (TPR1) and Son of Sevenless (SOS). Cell. Signal..

[32] Liou GY, Doppler H, Braun UB, Panayiotou R, Scotti Buzhardt M, Radisky DC (2015). Protein kinase D1 drives pancreatic acinar cell reprogramming and progression to intraepithelial neoplasia. Nat. Commun..

[33] Clark AR, Toker A (2014). Signalling specificity in the Akt pathway in breast cancer. Biochem. Soc. Trans..

[34] Hinz N, Jucker M (2019). Distinct functions of AKT isoforms in breast cancer: A comprehensive review. Cell Commun. Signal.

[35] Toker A, Yoeli-Lerner M (2006). Akt signaling and cancer: Surviving but not moving on. Can. Res..

[36] Dey-Guha I, Alves CP, Yeh AC, Sole X, Darp R (2015). A mechanism for asymmetric cell division resulting in proliferative asynchronicity. Mol. Cancer Res..

[37] Okawa ER, Gupta MK, Kahraman S, Goli P, Sakaguchi M, Hu J (2021). Essential roles of insulin and IGF-1 receptors during embryonic lineage development. Mol. Metab..

[38] Isohashi F, Endo H, Mukai M, Inoue T, Inoue M (2008). Insulin-like growth factor stimulation increases radiosensitivity of a pancreatic cancer cell line through endoplasmic reticulum stress under hypoxic conditions. Cancer Sci..

[39] Rajbhandari N, Lin WC, Wehde BL, Triplett AA, Wagner KU (2017). Autocrine IGF1 signaling mediates pancreatic tumor cell dormancy in the absence of oncogenic drivers. Cell Rep..

