# Supplementary material for: Gα15 in early onset of pancreatic ductal adenocarcinoma
Source: Sci Rep. 2021 Jul 21;11:14922. doi: 10.1038/s41598-021-94150-3 (PMC8295279; doi:10.1038/s41598-021-94150-3)
Supplement: Supplementary file 1 — Supplementary Information. [file 41598_2021_94150_MOESM1_ESM.docx]

**Gα15 in early onset of pancreatic ductal adenocarcinoma**

Giulio Innamorati*^#1^, Thomas M. Wilkie*^2^, Giorgio Malpeli^1^, Salvatore Paiella^1^, Silvia Grasso^1^, Borislav Rusev^3^, Biagio Eugenio Leone^4^, Maria Teresa Valenti^5^, Luca dalle Carbonare^5^, Samuele Cheri^6^, Alice Giacomazzi^1^, Marco Zanotto^1^, Vanessa Guardini^1^, Michela Deiana^6^, Donato Zipeto^6^, Michela Serena^6^, Marco Parenti^4^, Francesca Guzzi^4^, Rita Teresa Lawlor^3^, Giovanni Malerba^6^, Antonio Mori^6^, Giuseppe Malleo^1^, Luca Giacomello^1^, Roberto Salvia^♮1^ and Claudio Bassi^♮1^.

^1^ Department of Surgical Sciences, Dentistry, Gynecology and Pediatrics, University of Verona, Verona, Italy

^2^ Pharmacology Department, UT Southwestern Medical Center, Dallas, TX, USA

^3^ARC-Net Research Centre, University and Hospital Trust of Verona, Verona, Italy

^4^ Department of Medicine and Surgery, University of Milano-Bicocca, Monza, Italy

^5^ Department of Medicine, University of Verona, Verona, Italy

^6^ Department of Neurosciences, Biomedicine and Movement Sciences, University of Verona, Italy

* co-first authors, ^♮^ co-last authors,

^#^ corresponding author:

Giulio Innamorati giulio.innamorati@univr.it

Department of Surgical Sciences, Dentistry, Gynecology and Pediatrics,

University of Verona, c/o GB Rossi General Hospital

P.le L.A. Scuro 37134 Verona, Italy

Running title:

**Gα15 supports early PDA oncogenesis**

**Supplementary material**

*Integrative Bioinformatics analysis*

Assays for expression status of the *GNA15* gene include mRNA expression levels, gene methylation levels, and DNA sequence variants. Each readout was investigated for correlations with pancreatic cancer and healthy cells. The following public data repositories or resources were used:

1) GTEx Portal (<https://gtexportal.org/home/gene/GNA15>; January 2019), to study *GNA15* mRNA expression in normal human tissues.

2) provisional dataset for Pancreatic Cancer (PAAD, https://portal.gdc.cancer.gov/projects/TCGA-PAAD) of The Cancer Genome Atlas (TCGA, https://www.cancer.gov/about-nci/organization/ccg/research/structural-genomics/tcga) queried through the cBioPortal ((1), https://www.cbioportal.org/), to study *GNA15* mRNA expression in affected human tissues.

3) Broad Institute Cancer Cell Line Encyclopedia (CCLE(2), https://portals.broadinstitute.org/ccle), to study *GNA15* mRNA expression, DNA methylation and their correlation in human cancer cell lines.

4) Data from three mRNA microarrays studies, to study *GNA15* gene expression in PDA and healthy tissue(3-5).

5) The Blueprint Epigenome Program (http://www.blueprint-epigenome.eu/), to study the correlation between *GNA15* mRNA expression levels and methylation at single base level in leukemic cells lineages. Gα protein mutation was analyzed in the GRCh38 Cosmic database v88(6) on February 2019. Those who carried out the original analysis and collection of the data bear no responsibility for the further analysis or interpretation of it.

6) TCGA dataset LAML by MEXPRESS supported by the Common Fund of the Office of the Director of the National Institutes of Health, and by NCI, NHGRI, NHLBI, NIDA, NIMH, and NINDS.

*Gene expression of GNA15 co-expressed genes in human PD lesions.*

PAAD derived data were validated on total RNA extracted from xenografted human PDA lesions using TRIzol reagent (Life Technologies) following manufacturer’s instruction and quantified using Nanodrop (Thermo Fisher Scientific).

1 µg RNA was reverse-transcribed with High Capacity cDNA Reverse Transcription Kit (4368814) following manufacturer’s instruction and 1/10 of the reverse transcription was subjected to qPCR reactions.

mRNA was quantified by TaqMan assay (Applied Biosystems Life Technologies, Carlsbad, CA) performed in an ABI Prism 7900 HT instrument using the Universal PCR Master Mix (4304437) and assays for *GNA15* (Hs00157720_m1).

All PCR reactions contained primers and probe diluted 1:20 and 5 ng cDNA (total RNA equivalent) in 25 μl total volume, and samples were analyzed in triplicate. Thermal cycling included an initial 95 °C for 10 min then 40 cycles of 15 s at 95 °C for denaturation and 1 min at 60 °C for extension. As endogenous reference, *GAPDH* (glyceraldehyde-3-phosphate dehydrogenase) (Hs99999905_m1) and *ACTB* (beta-actin) (Hs99999903_m1) transcript levels were assayed in parallel. The relative mRNA expression of each sample was calculated by the standard curve method according to Lai et al(7).

*Cell Culture*

PT45(8) were mycoplasma tested every 2 months (B39132 Biotool Bimake.com) cultured in DMEM (10938025) supplemented with 10% fetal calf serum (FCS 10270106) and 1% pen-strep (15140122) all from Thermo Fisher Scientific. Doxycycline inducible cells were treated every 48 h with doxycycline (D9891-1G Sigma-Aldrich, St. Louis, MO, USA) unless otherwise noted. Cells were maintained at 37 °C in a humidified incubator with 5% CO2. Cell culture medium was changed every 3–5 days depending on cell density. For routine passage, cells were split at a ratio of 1:5–10 when they reached 85% to 90% confluence.

*Lentiviral Particles Production and Cell Transduction*

Reagents for replication-deficient lentivirus were from Sigma-Aldrich. Lentiviral particles were produced by transient transfection of 3 µg of pLKO_IPTG1xLacO (SHGLY-NM) containing previously validated shRNA sequences(8) and 3 µl of packaging mix (SHP001) into HEK293T cells in a 35 mm dish with Lipofectamine 2000 reagent (11668019, Life Technologies, Carlsbad, CA, USA), according to the manufacturer’s instructions. Viral supernatants were collected at 48 h, passed through a 0.2 μm filter and added to PT45 cells in the presence of 4 μg/mL hexadimethrine bromide (H9268) for 6 h followed by puromycin (P9620) selection 48 h post-infection.

*CRISPR/Cas9 Knockout*

CRISPR-mediated *GNA15* knockout was performed as previously described(9). Briefly, Lipofectamine 2000 was used to transfect HEK293T cells, as above, with pSpCas9(BB)-2A-Puro(PX459)V2.0 (Addgene #62988) containing guides designed in CHOPCHOP and CRISPR design software and targeting GNA15 gene in the first (462-AGGATGAGAAGGCCGCCGCCCGG and 897-CGGCCTTCTATCCTCCGTCAGG), and second (GTGCTCTTCCCGCTCTCGCCTGG) exons. Isolation of clonal cell lines was achieved with puromycin selection the third day after transfection followed by cloning into 96-well dishes. DNA was isolated from single clones and 3 independent clones were selected for displaying premature stop codons in *GNA15* sequence.

*Migration Activity*

Migration activity was evaluated by wound healing assay. Briefly, 6.0 × 10^5^ cells were seeded in each well of a 6-well plate. After 24 hours, straight parallel scratches were made using pipette tips. Cells were gently washed with phosphate-buffered saline (PBS) before adding 2 ml of fresh complete medium. Photographs of at least three different points were taken immediately and every hour for the following 72 hours by automatic microscopy (EVOS FL Auto, Thermo Fisher Scientific). The ImageJ wound healing tool plugin has been used to measure wound opening.

*Invasion Activity*

Invasion activity was evaluated by Boyden chamber assay, 10^4^ cells were seeded in duplicate in the upper chamber of a Transwell device, equipped with 8-μm microporous membranes (3422 Corning Inc., New York, NY) coated in the upper side with 1 mg/mL of growth factor reduced matrigel (11553620 Fisher Scientific) to simulate the ECM. The upper and lower compartments were filled with DMEM 10% FCS and cells were incubated at 37^o^C in 5% CO2 for 8 hours, transwells were next fixed in 100% methanol and stained with hematoxylin and eosin. Invasion activity was assessed in each membrane at the bottom side by counting the number of cells in 10 randomized microscopic fields (x100).

*Western Blot Analysis*

Whole cell protein extracts were prepared in PBS 1% NP40, 0.1% sodium dodecyl sulfate supplemented with protease and phosphatase inhibitors (Sigma-Aldrich), and centrifuged at 4 °C for 10 min at 14,000 g. Protein concentration was measured by the Pierce BCA protein assay (Thermo Fisher Scientific, Waltham, MA, USA). 30–40 µg of proteins were separated on 10% SDS-polyacrylamide gel electrophoresis gels and transferred to PVDF membrane. Membranes were blocked with 5% nonfat dry milk in Tris-buffered saline Tween-20 (TBST) for 1 h at room temperature and incubated with primary antibodies in 5% nonfat dry milk or bovine serum albumin (BSA) in TBST at 4 °C overnight followed by incubation with secondary antibody in 5% nonfat dry milk in TBST for 1-2 h at room temperature.

LumiLong (T-Pro Biotechnology) was used for protein detection.

Kinases activation was assessed with highly validated primary antibodies from Cell Signalling Technologies panAKT (4691S), pAKT S473 (4058S), pAKT S308 (2965S), pPKD1 S738,p742 (2054), pPKD1 pS910 (2054), pP38 T180,Y182 D3F9 (4511), pGSK α/β S21,S9 D17D2 (8566) but pERK42/44 T183 (Sigma-Aldrich M7802).

Gα15 expression was assessed in utilizing an antibody purchased from antibodies online (ABIN110590). The band corresponding to the endogenous protein was carefully verified(8) over a significant background. Extended version of the cropped images are shown in Supp. Fig. S10-12.

**Supplementary table and figures**

|  | **Whole organism** | | | **In the pancreas** | | |
| --- | --- | --- | --- | --- | --- | --- |
| **Gene** | **Total # of unique samples** | **Unique samples with mutations** | **Hotspot mutations**  (Q209, R183)* | **Mutation frequency** | **Hotspot mutations**  (Q209, R183)* | **Aminoacid missense substitution** |
| ***GNAQ*** | 73848 | 1043 | 710 | 2/3052 | 0 | T96S, R166C |
| ***GNA11*** | 68088 | 709 | 404 | 1/2915 | 0 | I141N |
| ***GNA14*** | 47610 | 117 | 0 | 6/1815 | 0 | A19V, I52M, I37M, P170S, P294L, D342Y |
| ***GNA15*** | 47318 | 140 | 4 | 2/1719 | 0 | I94M, R145Q |

Supplementary table 1 - Activating mutations of Gα subunits belonging to the Gq class.

A search of the Cosmic database for driver mutations in Gq class alpha subunit.

Most activating mutations are located in position Q209 of *GNAQ* and *GNA11* and are found in cutaneous and ocular melanoma(10). Four *GNA15* activating alleles were identified at R186H: one glioblastoma, two lung squamous carcinoma and a head and neck squamous carcinoma. The analysis included neoplasia arising in several tissues, including bone marrow and skin, where *GNA15* expression is relatively high. In pancreas, only a few scattered amino acid missense substitutions were reported (far right column).

*Numbering is based on *GNAQ/11* amino acid sequence.

Supplementary table 2 - Demographic characteristics of the cases described in fig 4 C

**
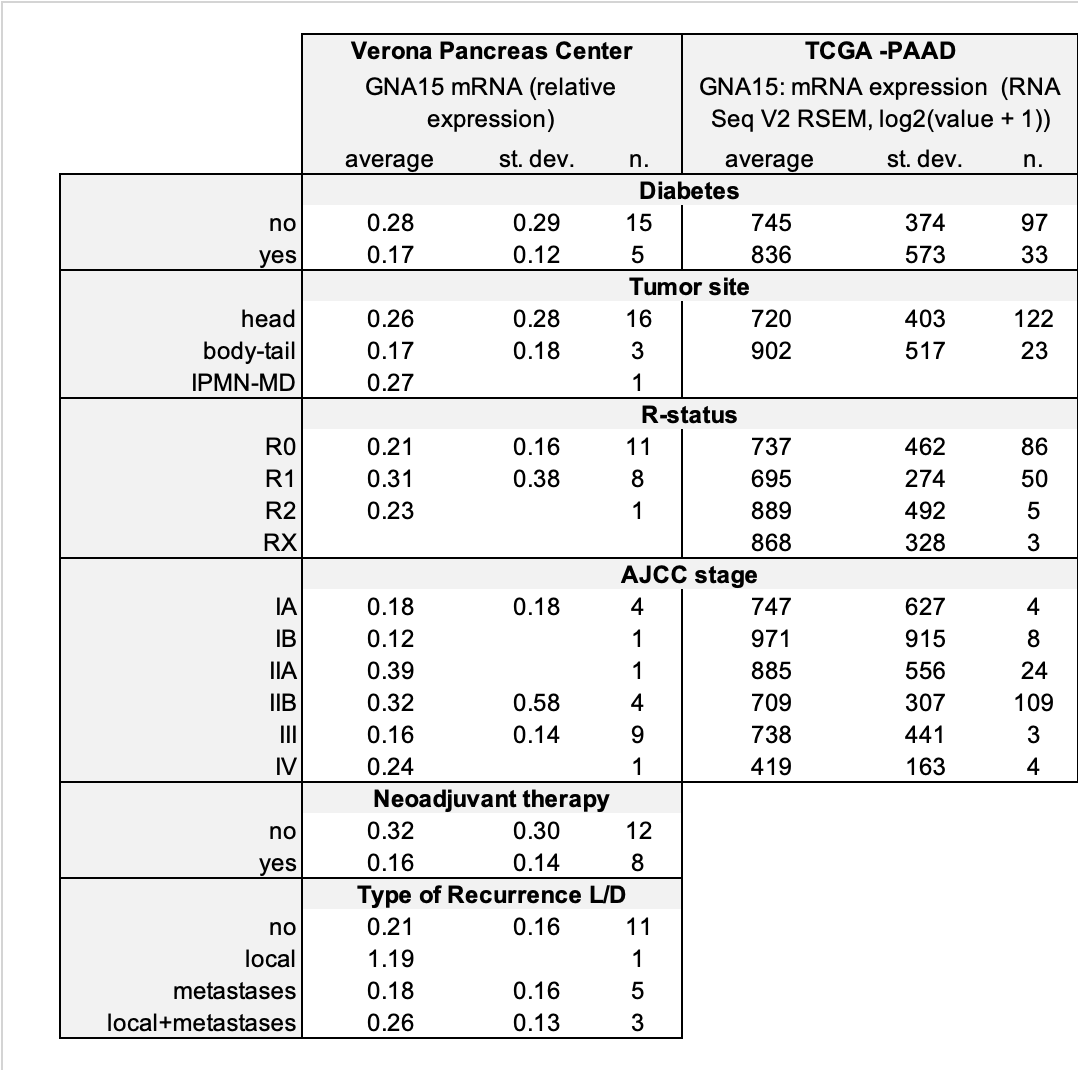
**

Supplementary table 3 – *GNA15* expression level stratified based on the clinical characteristics.

For the cases described in fig 4 C and present in the PAAD dataset, *GNA15* expression level is detailed for each subgroup defined by clinical characteristics specified in the left column.


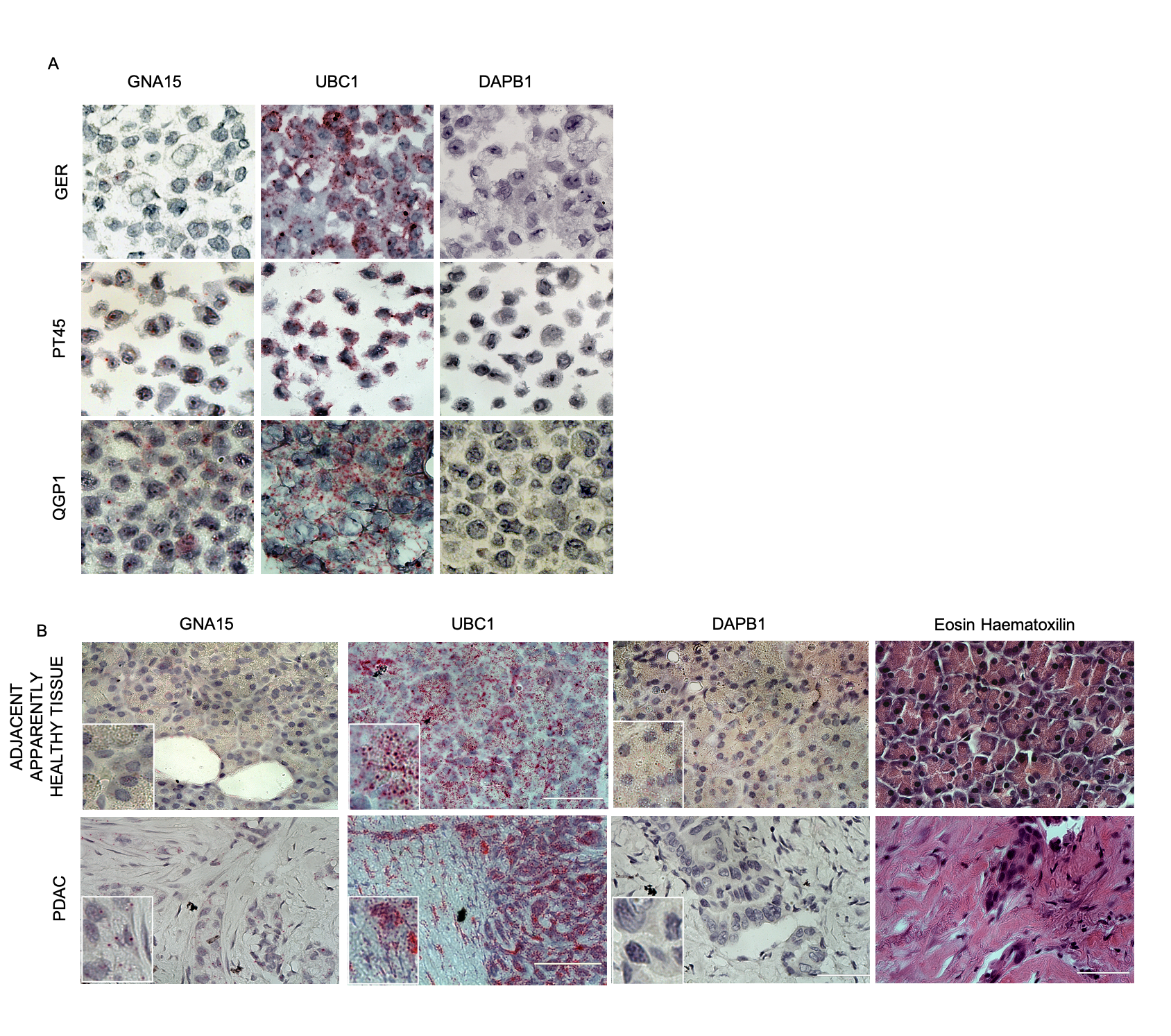


Supplementary figure S1 - **Validation of *GNA15* probe for ISH.**

Transformed pancreas samples were analyzed by ISH rather than immunohistochemistry of endogenous Gα15, due to poor specificity in IHC of all antibodies tested (data not shown).

**A)** Pancreatic cancer cells previously described for not expressing (GER) or expressing (PT45, QGP1) GNA15, were fixed and included in agarose first and in paraffin later. 5µm slices were hybridized to specific probes for *GNA15*, for a positive (UBC) and a negative (DAPB1) control as indicated.

**B)** FFPE from PDAC and adjacent apparently healthy tissue analyzed for *GNA15* expression, positive and negative control by ISH as indicated. Right panels show eosin haematoxylin staining of slices from the same blocks.


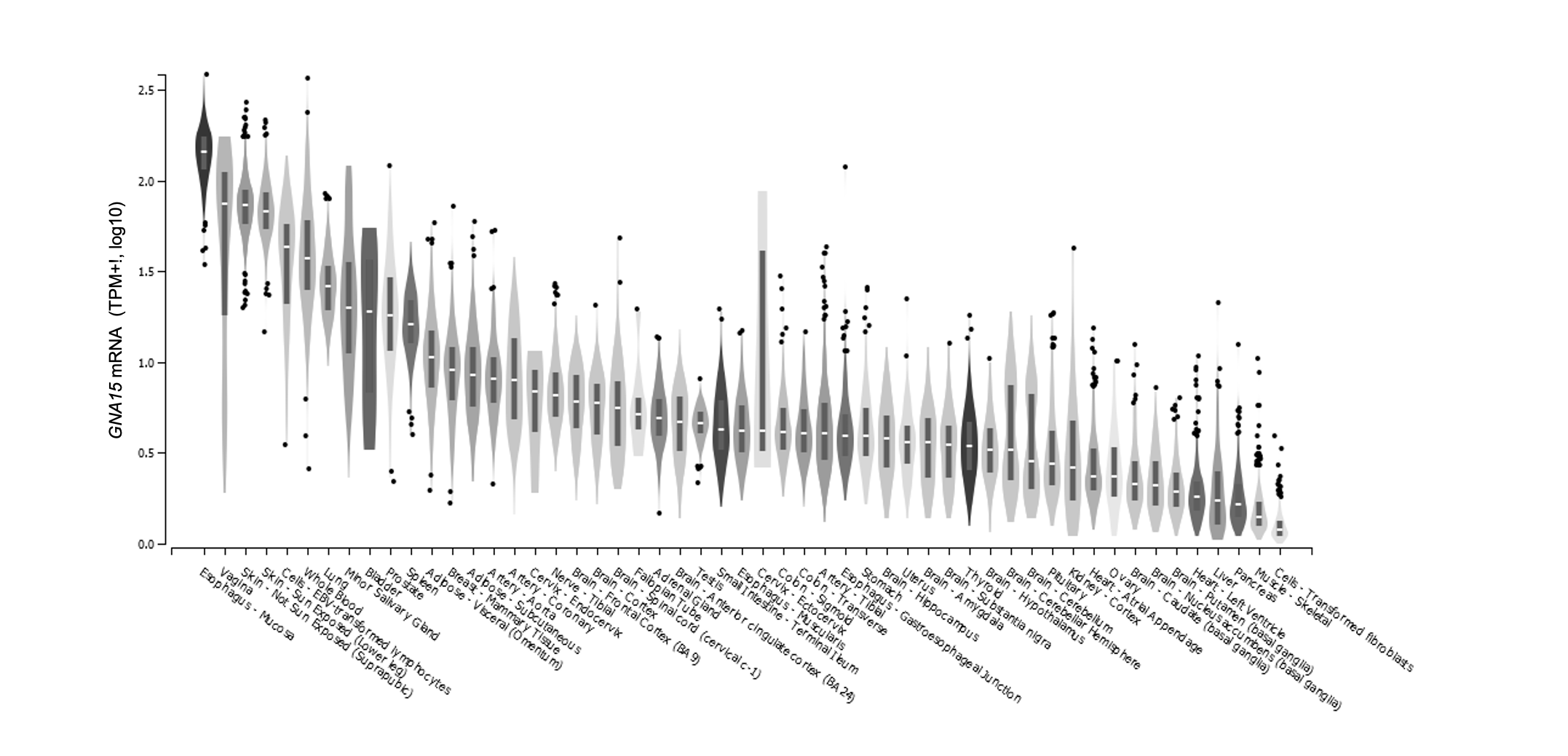


Supplementary figure S2 - ***GNA15* mRNA expression in human tissues according to the GTEx.** Values shown in TPM (transcripts per million reads). Box plots are shown as median and 25th and 75th percentiles: points are displayed as outliers if they are 1.5 fold above or below the interquartile range.


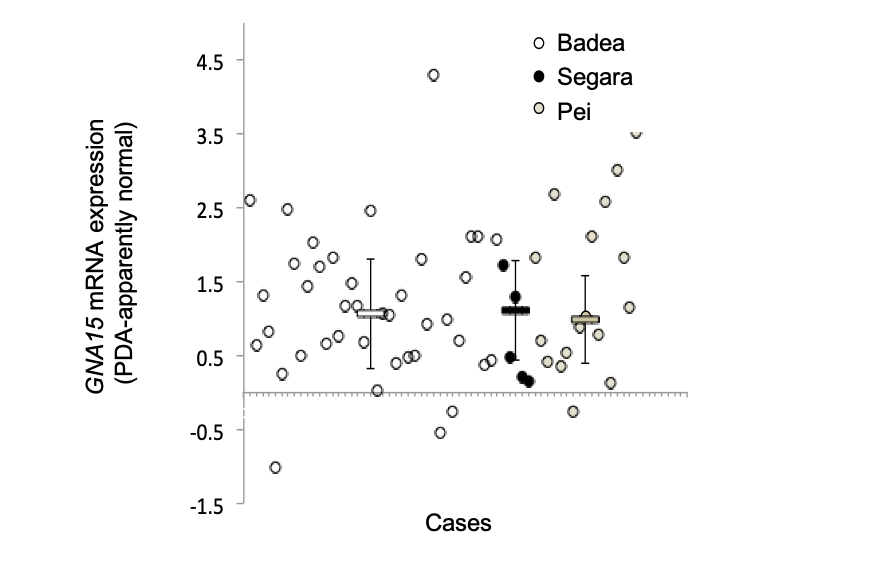


Supplementary figure S3 - Follow-up correlations with *Gα subunits* mRNA expression level.

*GNA15* mRNA levels in PDA tissue vs matched adjacent healthy tissue analyzed by microarray in 3 published datasets. For each paired case available, *GNA15* expression value in PDA is plotted after subtracting its respective value in adjacent healthy tissue; therefore, all cases falling above the abscissa overexpress *GNA15*. The bars indicate the average ± absolute deviation for each reference. The three studies (Pei et al.(5) Badea et al.(3) Segara et al.(4)) utilized Human Genotype U133 arrays.


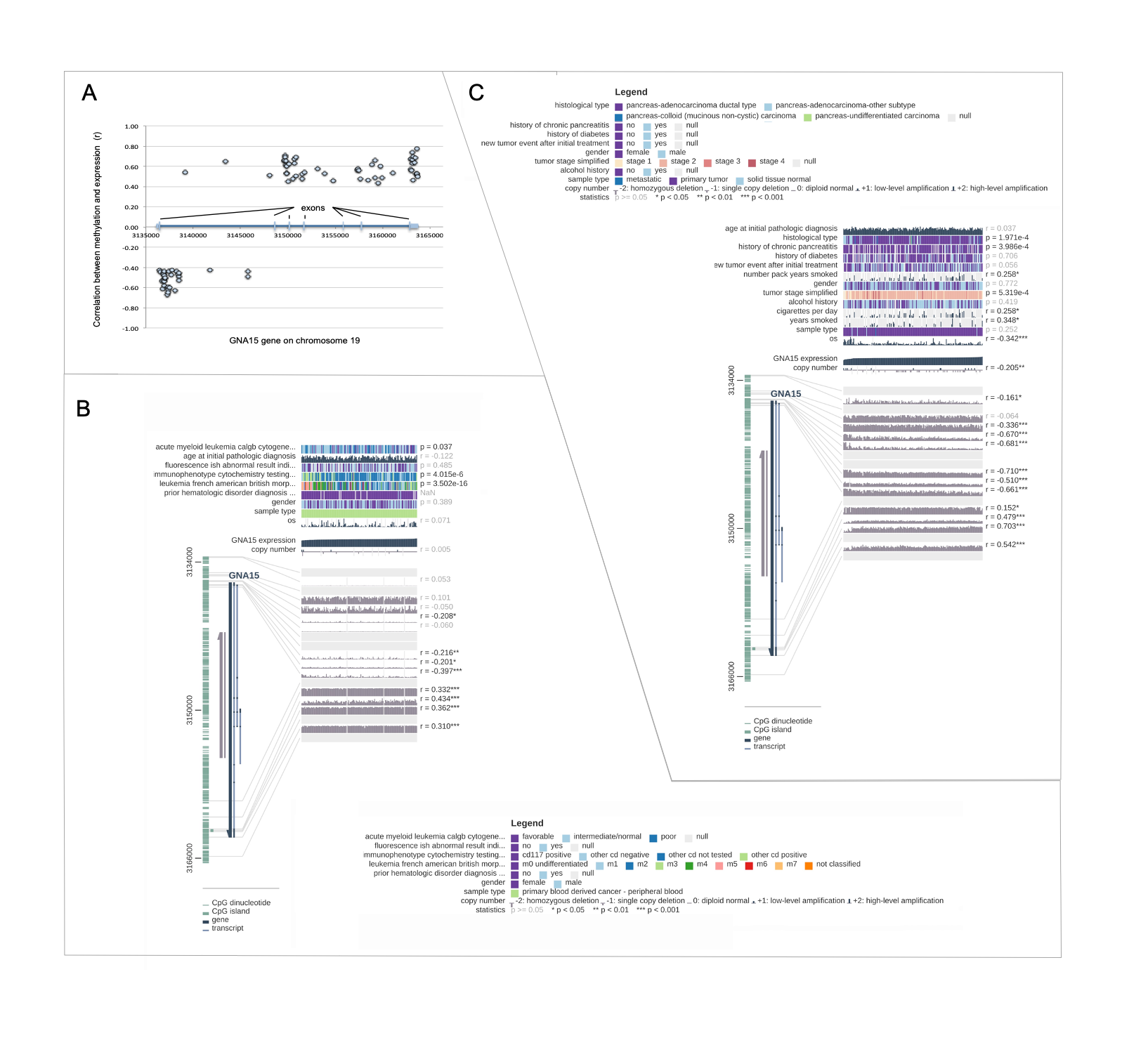


Supplementary figure S4 - **Analysis of *GNA15* gene expression and methylation in PDAC and Leukemias.**

**A)** Analysis of single CpGs on *GNA15* gene in leukocytes and hematopoietic cell types from data released by the Blueprint Epigenome Program (<http://www.blueprint-epigenome.eu/>).

Two regions of differential methylation were identified. The methylation level of most CpGs correlated inversely with gene expression across a large region encompassed the promoter, the transcription start site, the first exon and part of the first intron. By contrast, hypermethylated CpGs in a region 3’ of *GNA15* correlated with increased gene expression.

**B)** Analysis of *GNA15* gene expression and methylation based on the LAML (AML) dataset of the TCGA performed in MEXEXPRESS(11) (176 samples) showed similar to A) hypomethylation around the promoter in 4 CPGs and hypomethylation of 4 CPGs at the 3’ end of *GNA15*

**C)** Analysis of *GNA15* gene expression ad methylation based on the PAAD dataset of the TCGA performed in MEXPRESS (183 samples).


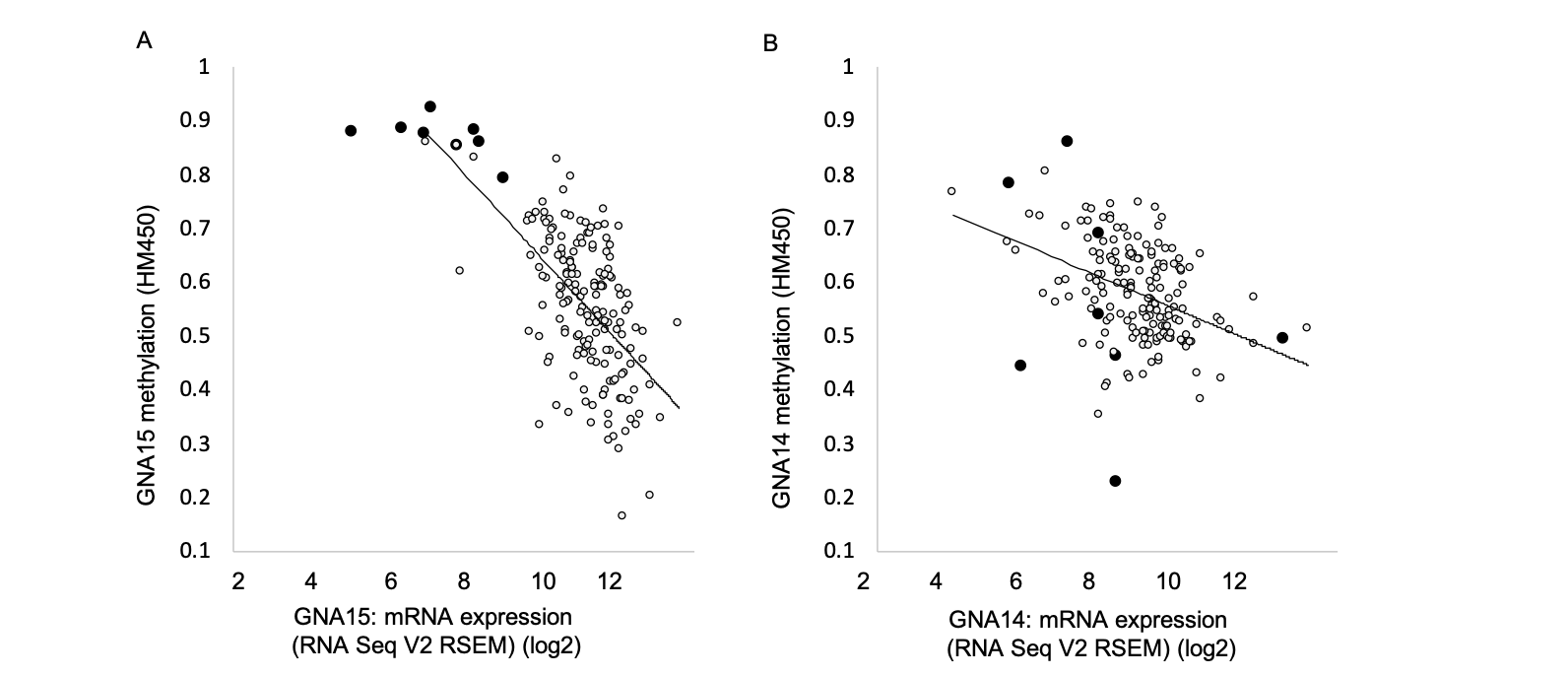


Supplementary figure S5 – **Direct comparison of gene expression and methylation in PDAC vs NET.**

**A)** In TCGA, methylation analysis is available for 178 PDA (light gray dots) and 8 NETs (black dots). The methylation level around *GNA15* is plotted vs. mRNA expression (Spearman’s -0.59, p=2.25 e^-18^. R^2^=0.43).

**B)** The methylation level around *GNA14,* analyzed as A, was plotted vs. mRNA expression (Spearman’s -0.38, p=1.17 e^-7^. R^2^=0.14). Light gray dots are PDA and black dots are NETs.


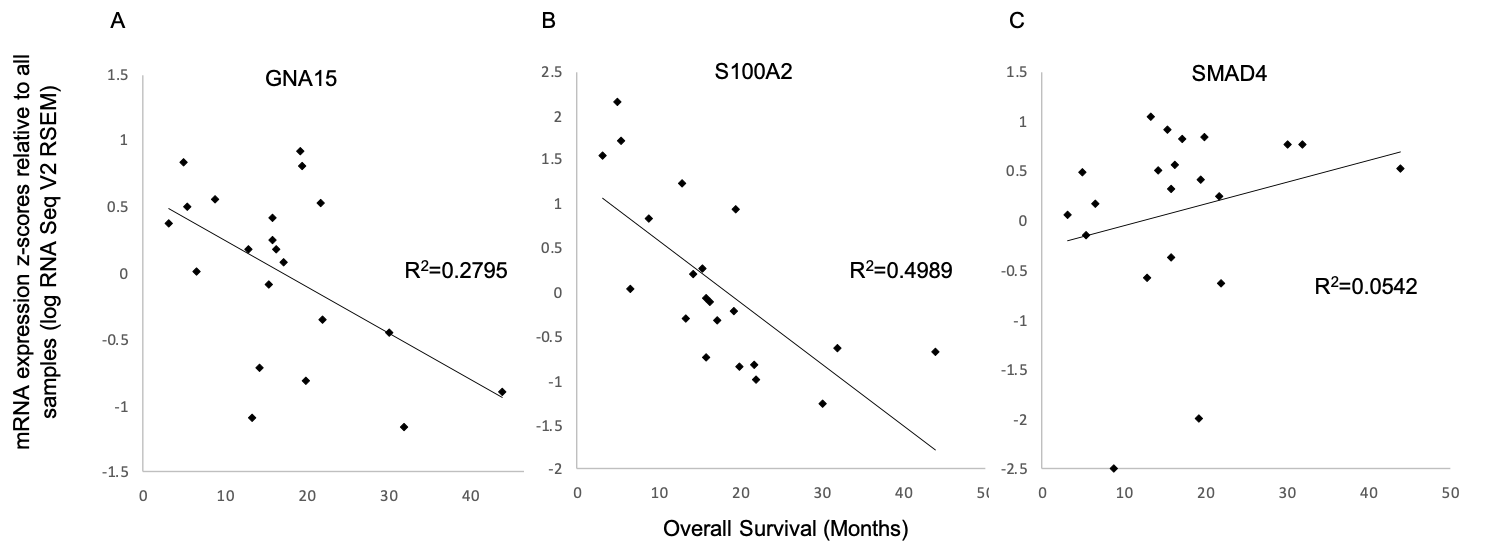


Supplementary figure S6 – **Expression of** **potential prognostic biomarkers in PDA tissue from patients initially diagnosed by cytology in the TCGA-PAAD cohort**

A) The expression levels of *GNA15* mRNA plotted vs. overall survival (Pearson = -0.53, p=0.0137).

B) The expression levels of *S100A* mRNA plotted vs. overall survival (Pearson = -0.71, p=3,415 e^-4^).

C) The expression levels of *SMAD4* mRNA plotted vs. overall survival (Pearson = 0.23, p=0.310).


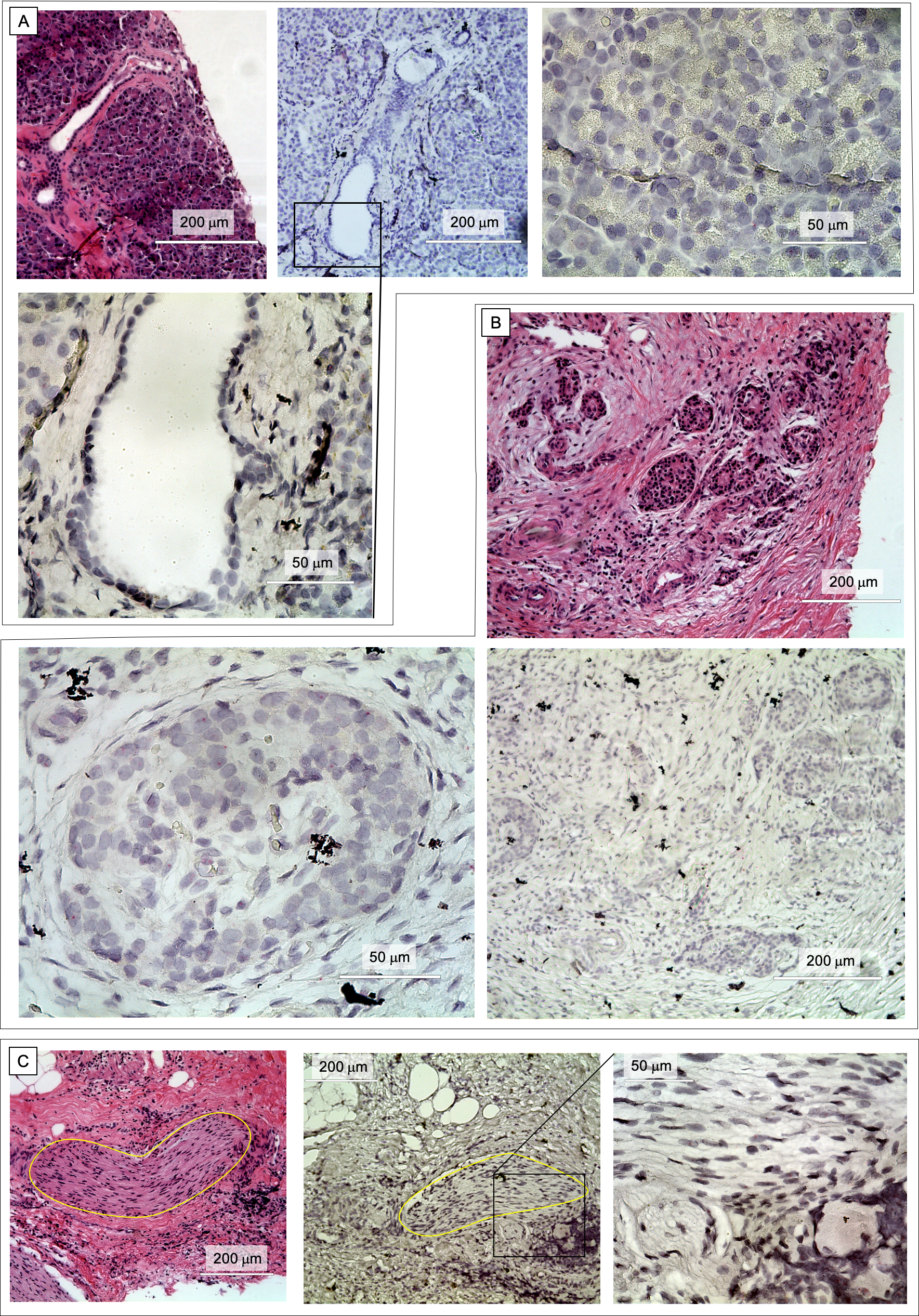


Supplementary figure S7 – ***GNA15* gene expression in pancreas components analyzed by ISH.**

In the first micrograph of each panel eosin haematoxylin stained sections, all other panels are ISH utilizing *GNA15* probe. *GNA15* mRNA is absent in all the normal components or in the stroma: (**A)** a duct and acini, **(B)** islets, **(C)** a nerve.


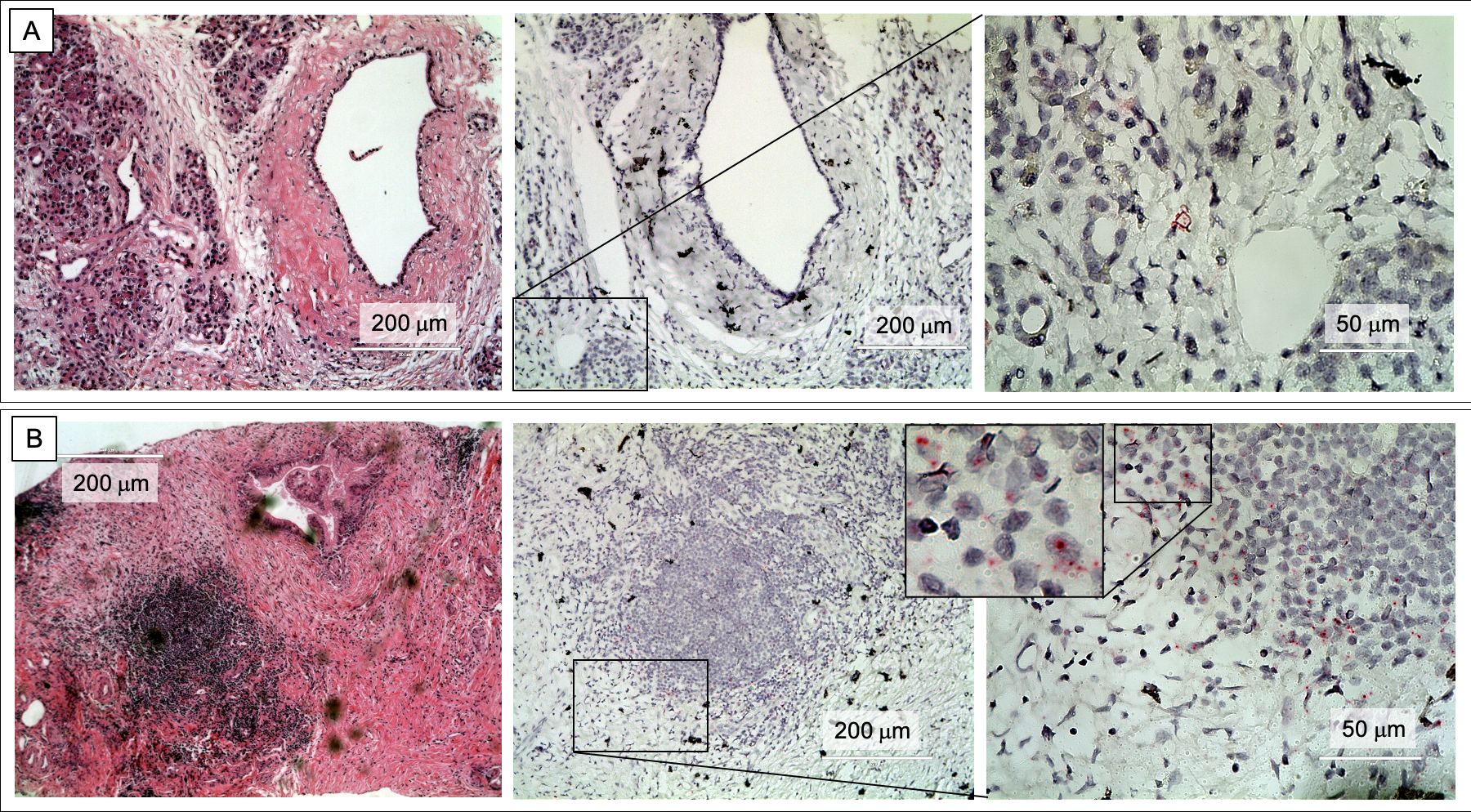


Supplementary figure S8 – ***GNA15* gene expression in pancreatic dysplasia analyzed by ISH.**

In the left micrographs eosin hematoxylin stained sections, all other panels are ISH utilizing *GNA15* probe. **(A)** Areas of pancreatic tissue displaying pancreatitis or low grade dysplasia were negative. **(B)** Occasionally, cells positive for *GNA15* expression were found in foci of infiltrating lymphocytes were found.


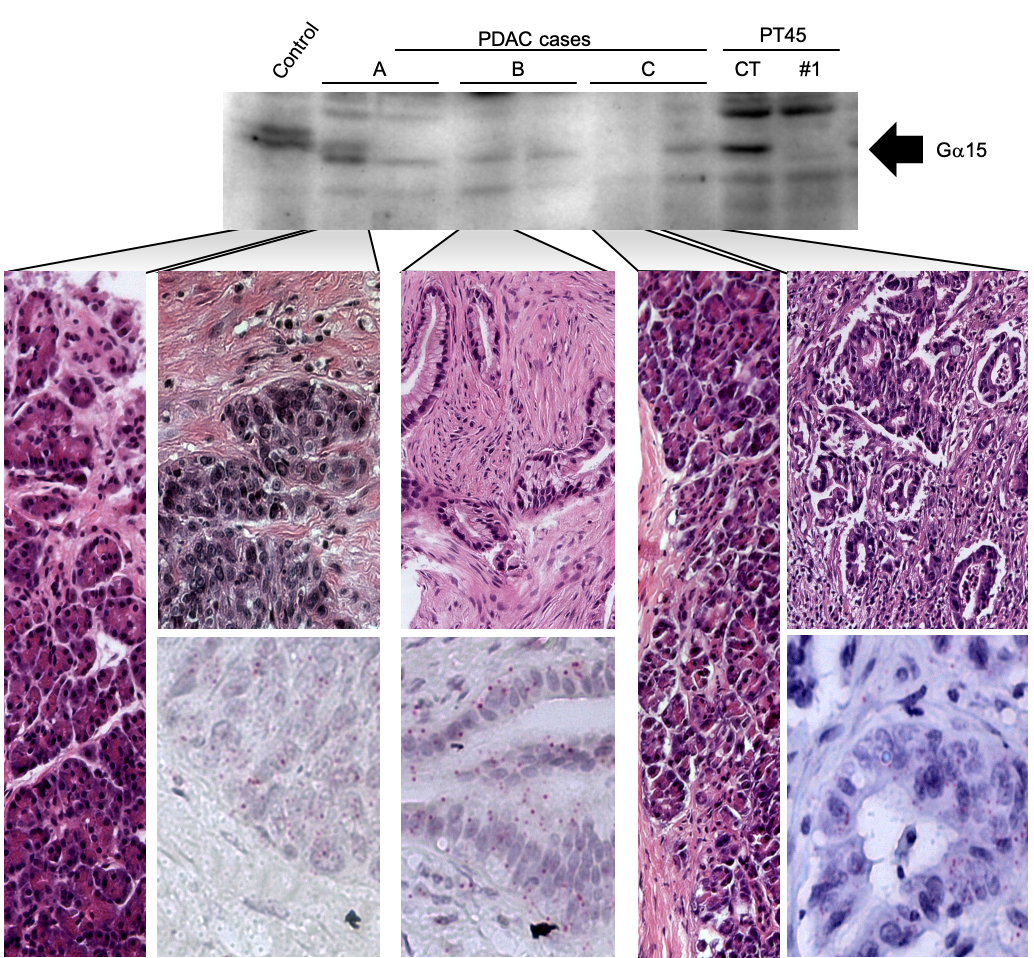


Supplementary figure S9 – ***Gα15* protein expression in PDA.**

Human pancreas biopsies were analyzed by western blot for Gα15 expression. Extended version of the cropped images is shown in Supp. Fig. S14.The band corresponding to Gα15 is indicated by the arrow and confirmed in the last two lanes where WT PT45 cells (CT) are compared to a *GNA15* CRISPR/CAS9 knoc-kout clone (#1). In the first lane (control) a sample of an area adjacent to apparently healthy tissue obtained from a pancreas affected by chronic pancreatitis. The left lane of C was also adjacent to apparently healthy tissue but collected from a PDA patient. All the other tissue fragments examined were selected for displaying numerous lesions in the adjacent area analyzed by eosin hematoxylin and for expressing high levels of mRNA according to ISH (>.3 in the scale of panel S) as shown in the representative micrographs below.


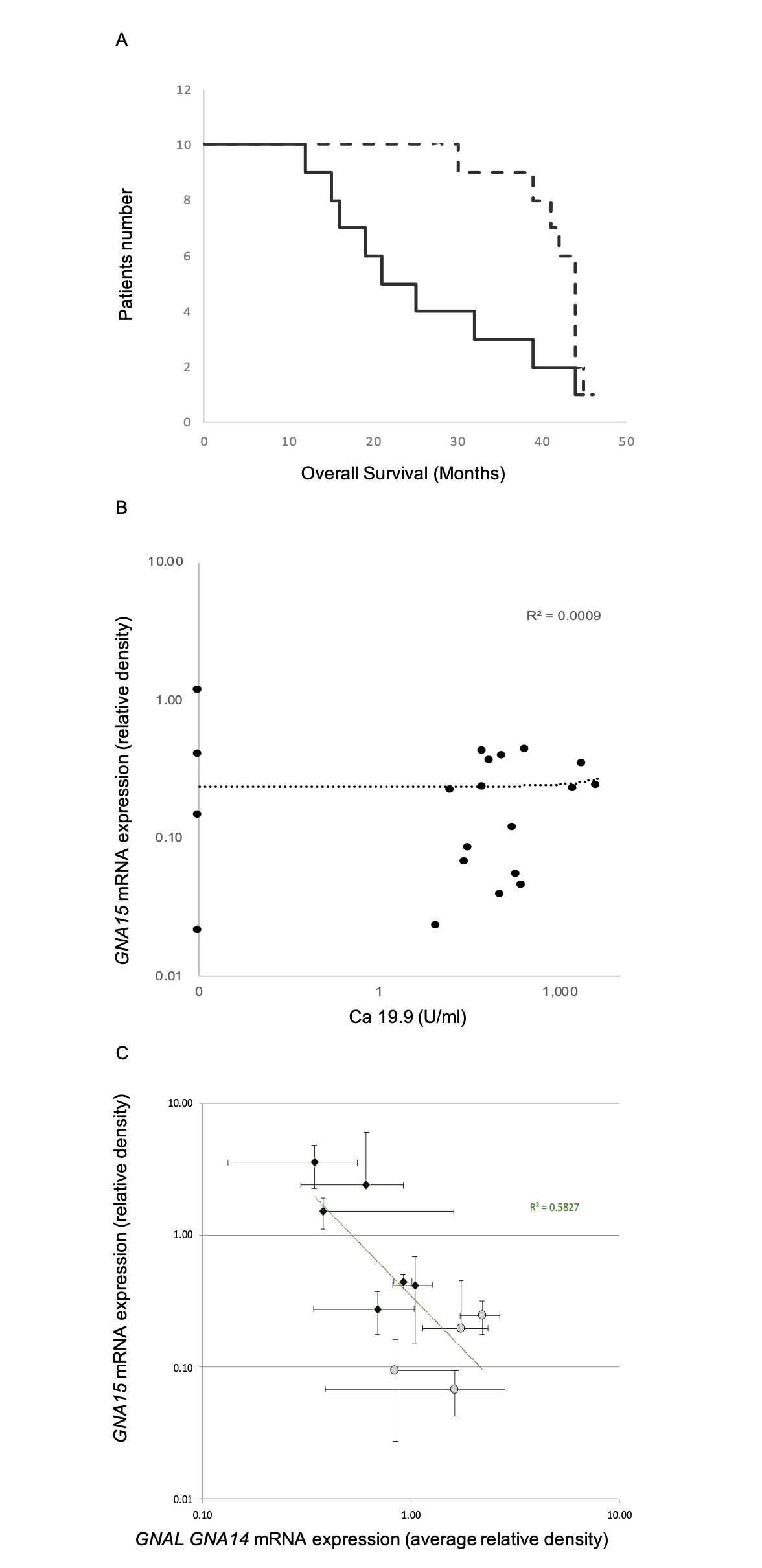


Supplementary figure S10 – **Estimate of the prognostic value of** ***GNA15* expression in the cohort of the Pancreas Institute of Verona**

A) The expression levels of *GNA15* mRNA (dots density in ISH) plotted vs. overall survival (Pearson = -0.608, p= 0.0044).

B) The expression levels of *GNA15* mRNA (dots density in ISH) plotted vs. Ca19.9 levels (Pearson = -0.066, p= 0.9529).

C) The mRNA expression levels (TaqMan RT-PCR) of *GNA15* plotted vs. the average of GNAL and GNA14. Black circles represent PDAC and IPMN in fig. 4E, gray diamonds the remaining controls.


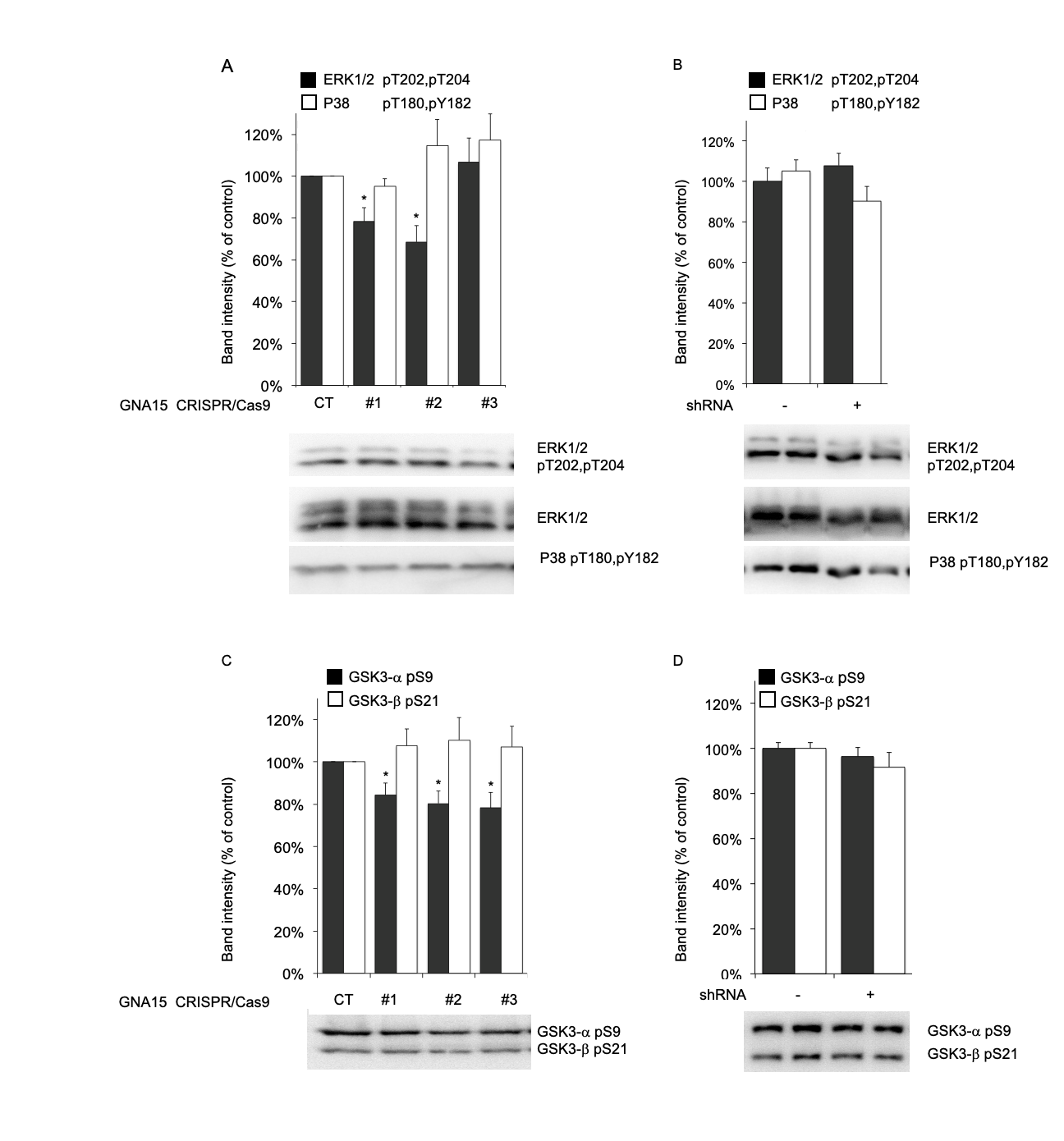


Supplementary figure S11 *-* Impact of Gα15 knock-down on the activation state of signaling kinases.

The impact of a reduction of Gα15 expression was evaluated by immunoblot analysis of phospho-amino acids diagnostic of the activation state of kinases central to PDAC cells physiology (mean ± SEM, * P<0.005, ** P<0.0001, *** P<0.0005). Extended versions of the cropped images are shown in Supp. Fig. S15.

A) ERK 1/2 phosphorylation (n=15) and P38 phosphorylation (n=12)

B) ERK 1/2 phosphorylation (n=10) and P38 phosphorylation (n=10)

C) GSK phosphorylation on (n=8)

D) GSK phosphorylation on (n=16)


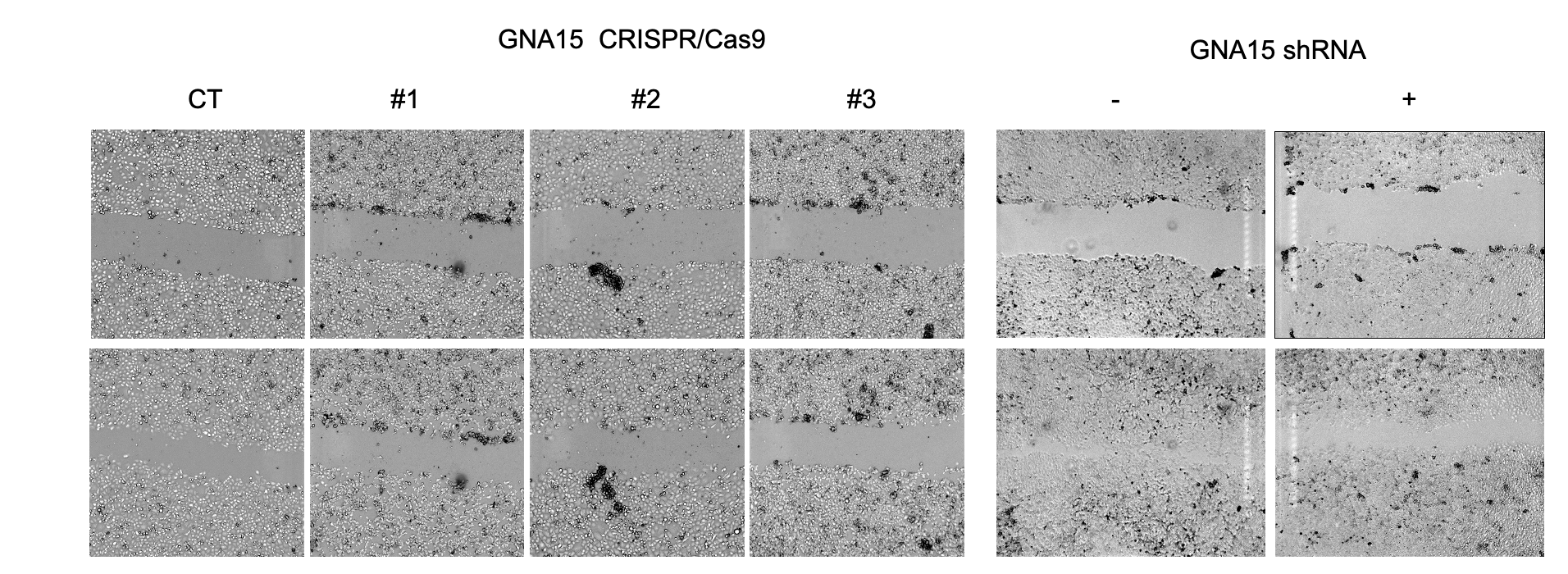


Supplementary figure S12 *-* Impact of Gα15 knock-down on cell migration.

Representative images of the scratch assay in fig. 5F documenting the impact of a reduction of Gα15 expression on cell migration. The initial images are in the upper panels, the final images are in the lower panels.

Supplementary figure S13 – **Expanded version of the western blot images in fig. 5**


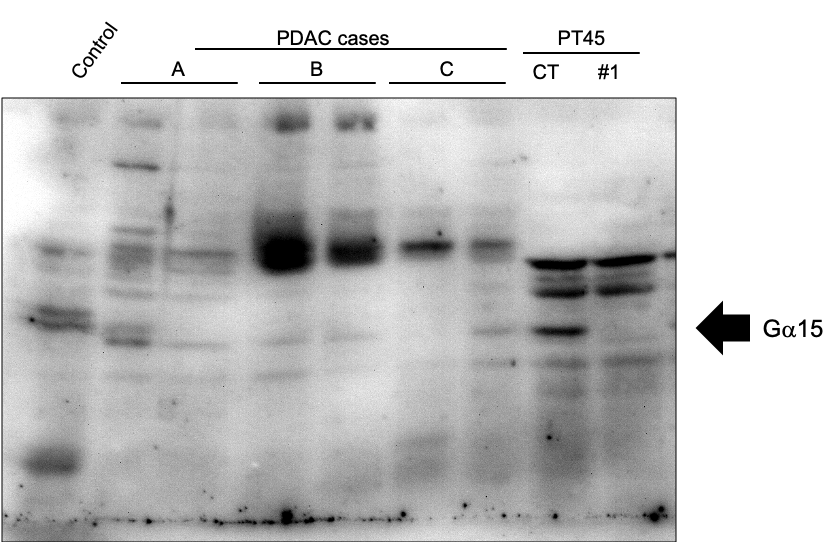


Supplementary figure S14 – **Expanded version of the western blot images in supplementary fig. S9.**

Supplementary figure S15 – **Expanded version of the western blot images in supplementary fig. S11.**

**REFERENCES**

1. Gao J, Aksoy BA, Dogrusoz U, Dresdner G, Gross B, Sumer SO*, et al.* Integrative analysis of complex cancer genomics and clinical profiles using the cBioPortal. Sci Signal **2013**;6:pl1

2. Safikhani Z, Smirnov P, Thu KL, Silvester J, El-Hachem N, Quevedo R*, et al.* Gene isoforms as expression-based biomarkers predictive of drug response in vitro. Nat Commun **2017**;8:1126

3. Badea L, Herlea V, Dima SO, Dumitrascu T, Popescu I. Combined gene expression analysis of whole-tissue and microdissected pancreatic ductal adenocarcinoma identifies genes specifically overexpressed in tumor epithelia. Hepatogastroenterology **2008**;55:2016-27

4. Segara D, Biankin AV, Kench JG, Langusch CC, Dawson AC, Skalicky DA*, et al.* Expression of HOXB2, a retinoic acid signaling target in pancreatic cancer and pancreatic intraepithelial neoplasia. Clinical cancer research : an official journal of the American Association for Cancer Research **2005**;11:3587-96

5. Pei H, Li L, Fridley BL, Jenkins GD, Kalari KR, Lingle W*, et al.* FKBP51 affects cancer cell response to chemotherapy by negatively regulating Akt. Cancer Cell **2009**;16:259-66

6. Sondka Z, Bamford S, Cole CG, Ward SA, Dunham I, Forbes SA. The COSMIC Cancer Gene Census: describing genetic dysfunction across all human cancers. Nature reviews **2018**;18:696-705

7. Lai J-P, Yang J-H, Douglas SD, Wang X, Riedel E, Ho W-Z. Quantification of CCR5 mRNA in human lymphocytes and macrophages by real-time reverse transcriptase PCR assay. Clinical and diagnostic laboratory immunology **2003**;10:1123-8

8. Giovinazzo F, Malpeli G, Zanini S, Parenti M, Piemonti L, Colombatti M*, et al.* Ectopic expression of the heterotrimeric G15 protein in pancreatic carcinoma and its potential in cancer signal transduction. Cellular signalling **2013**;25:651-9

9. Deiana M, Dalle Carbonare L, Serena M, Cheri S, Parolini F, Gandini A*, et al.* New Insights into the Runt Domain of RUNX2 in Melanoma Cell Proliferation and Migration. Cells **2018**;7

10. O'Hayre M, Vazquez-Prado J, Kufareva I, Stawiski EW, Handel TM, Seshagiri S*, et al.* The emerging mutational landscape of G proteins and G-protein-coupled receptors in cancer. Nature reviews **2013**

11. Koch A, De Meyer T, Jeschke J, Van Criekinge W. MEXPRESS: visualizing expression, DNA methylation and clinical TCGA data. BMC Genomics **2015**;16:636
